# Perylenetetracarboxylic Diimide Composite Electrodes as Organic Cathode Materials for Rechargeable Sodium-Ion Batteries: A Joint Experimental and Theoretical Study

**DOI:** 10.1021/acsomega.3c07621

**Published:** 2024-01-31

**Authors:** Sebastian Liebl, Josef M. Gallmetzer, Daniel Werner, Dogukan H. Apaydin, Thomas S. Hofer, Engelbert Portenkirchner

**Affiliations:** † Institute of Physical Chemistry, University of Innsbruck, 6020 Innsbruck, Austria; ‡ Institute of Materials Chemistry, Vienna University of Technology, 1060 Vienna, Austria; § Institute of General, Inorganic and Theoretical, Chemistry University of Innsbruck, 6020 Innsbruck, Austria

## Abstract

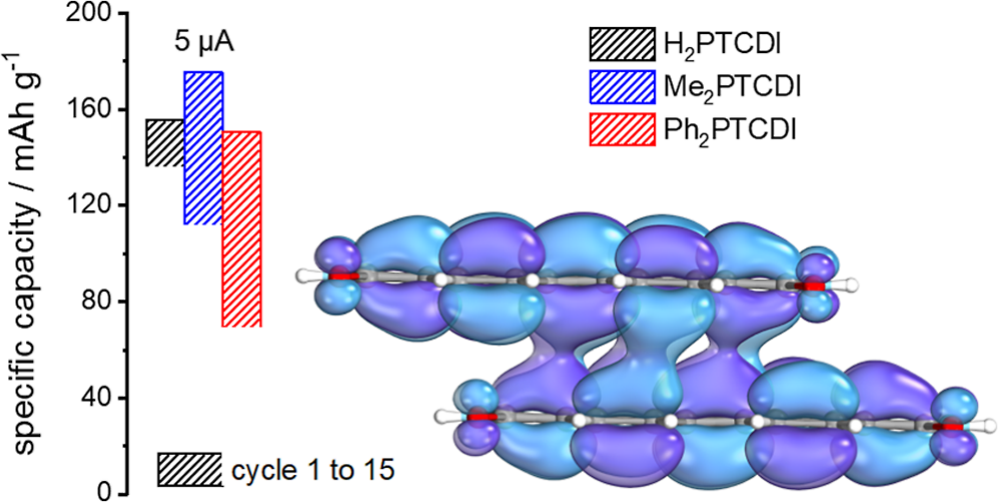

The organic semiconductor 3,4,9,10-perylenetetracarboxylic diimide (PTCDI), a widely used industrial pigment, has been identified as a diffusion-less Na-ion storage material, allowing for exceptionally fast charging/discharging rates. The elimination of diffusion effects in electrochemical measurements enables the assessment of interaction energies from simple cyclic voltammetry experiments through the theoretical work of Laviron and Tokuda. In this work, the two N-substituted perylenes, *N*,*N*′-dimethyl-3,4,9,10-perylenetetracarboxylic diimide (Me_2_PTCDI) and *N*,*N*′-diphenyl-3,4,9,10-perylenetetracarboxylic diimide (Ph_2_PTCDI), as well as the parent molecule 3,4,9,10-perylenetetracarboxylic diimide (H_2_PTCDI) are investigated as thin-film composite electrodes on carbon fibers for sodium-ion batteries. The composite electrodes are analyzed with Raman spectroscopy. Interaction parameters are extracted from cyclic voltammetry measurements. The stability and rate capability of the three PTCDI derivatives are examined through galvanostatic measurements in sodium-ion half-cell batteries and the influence of the interactions on those parameters is evaluated. In addition, self-consistent charge density function tight binding calculations of the different PTCDI systems interacting with graphite have been carried out. The results show that the binding motif displays notable deviations from an ideal ABA stacking, especially for the neutral state. In addition, data obtained for the electron-transfer integrals show that the difference in performance between different PTCDI thin-film batteries cannot be solely explained by the electron-transfer properties and other factors such as H-bonding have to be considered.

## Introduction

Renewable energy sources, like solar or wind power, play an important role in mitigating issues related to global warming.^1,2^ A disadvantage of these power sources is the discontinuous production of electricity due to intermittent environmental conditions like sunshine duration for solar power plants or variable wind conditions. Additionally, abundant renewable resources are often localized away from consumer centers.^3,4^ A reliable energy supply depends on electricity storage facilities.^5^ One possibility to store the excess capacity is to use rechargeable batteries. Ideally, these batteries possess high power and energy density while simultaneously being environmentally friendly and cheap in production.^6,7^ Organic electrode materials for alkali-ion batteries are a promising candidate to fulfill these criteria.^8^ The growing costs and limited resources of Li has shifted the scientific interest to Na-ion batteries (SIBs) due to the high natural abundance of Na.^9^ Small organic semiconducting molecules, as the core element for low-cost and energy-efficient “green” electrodes, are a promising material class for such SIBs.^10^ Organic molecules with carbonyl groups as their reactive moiety belong to this material class and have demonstrated great potential.^11,12^ The charge storage is facilitated via an ion-coordination mechanism of the Na ion to the negatively charged oxygen atom of the electrochemically reduced carbonyl group and its reversible dissociation during the reverse oxidation, demonstrated for anthraquinone and its derivatives.^13,14^ While most of these “carbonyl compounds” have fast Na-insertion kinetics and high capacities, they suffer from low electronic conductivity, high solubility in the electrolyte, and low potentials as cathodes. A strategy to tackle these disadvantages is to modify the underlying framework of the molecule to tune properties like redox potential or solubility.^15,16^ In 2015, Deng et al.^17^ investigated the parent perylene diimide molecule, 3,4,9,10-perylenetetracarboxylic diimide (H_2_PTCDI), which has a hydrogen residual on the nitrogen atom of the imide moiety, as cathode material for SIBs. The PTCDI molecule undergoes a reversible two-electron redox reaction in the potential window of 1–3 V vs Na/Na^+^. It shows a stable charge/discharge capacity of 138.7/138.6 mAh g^–1^ (under a constant current of 10 mA g^–1^) after the first cycle of a galvanostatic cycle experiment, which correlates to a two-electron redox reaction per PTCDI molecule (having a theoretical capacity of 137 mAh g^–1^ for a two-electron redox reaction).^17^ The PTCDI molecule, with its perylene core and the two imide groups as redox active moieties, forms a conjugated system, which is ideal for structural modification possibilities. On the one hand, the perylene core can be modified on its four bay and four ortho positions via the replacement of the hydrogen atoms by other atoms or molecules, and on the other hand, the residual on the nitrogen atom of the imide group is highly interchangeable. In 2017, Banda et al.^18^ modified the bay positions of the perylene core by replacing the hydrogen atoms one by one with bromine. With each replacement the electron-withdrawing ability of the bromine atoms shifted the first and second reduction potentials of the PTCDI molecule to higher voltages. The potential difference between the first reduction of the unsubstituted PTCDI molecule and the four-times substituted PTCDI was measured as 130 mV in a cyclic voltammetry (CV) experiment. Furthermore, they showed that the replacement of the hydrogen atoms with bromine twisted the perylene core, resulting in a reduced potential difference between the first and second reduction reaction. For the three- and four-times substituted PTCDI molecule, the two-step reduction merged into one, giving rise to a concerted two-electron redox reaction.^18^ This shows that physicochemical properties as well as the geometry of the molecules play an important role in the electrochemical characteristics.

In a very recent publication, Huang et al.^12^ were able to show that H_2_PTCDI serves as an exceptionally well-suited material for long lifespan and high-energy aqueous organic batteries. Our group also investigated the H_2_PTCDI molecule as a thin film on a carbon fiber substrate as an electrode for SIBs.^19^ H_2_PTCDI on carbon fibers proved to be beneficial over conventional substrates, like carbon-coated copper, demonstrating higher charge-transfer kinetics due to a lower pore resistance.^20^ The PTCDI molecules were found to be adsorbed planar onto the graphite-like substrate fibers forming a stacked film which is stabilized by π–π interactions between the layers and substrate and strong intermolecular H-bonding. This gave rise to a diffusion-less mechanism, suggesting that the diffusion of Na ions into the film is exceptionally fast.^19^ In this work, by exchanging the hydrogen residuals on the nitrogen atoms of the imide moieties with methyl and phenyl groups, the two additional PTCDI derivatives *N*,*N*′-dimethyl-3,4,9,10-perylenetetracarboxylic diimide (Me_2_PTCDI) and *N*,*N*′-diphenyl-3,4,9,10-perylenetetracarboxylic diimide (Ph_2_PTCDI) are investigated and compared to the parent H_2_PTCDI molecule. The molecular structure and the proposed redox reaction of the PTCDI molecules are depicted in Figure S1. Additionally, theoretical calculations at self-consistent charge density function tight binding (SCC DFTB) have been performed to investigate the initial absorption steps relevant for the thin-film formation on the graphite carrier, providing insight into the physicochemical properties of the PTCDI molecules in the cathode material. As done in a previous study,^14^ the basin-hopping minimization strategy has been employed to investigate the binding motifs of the PTCDI molecules on a model four-layer graphite system. The relevant conformations have been examined for both, the neutral and the 2-fold sodiated states of the target molecules H_2_PTCDI, Me_2_PTCDI, and Ph_2_PTCDI, respectively. Additionally, harmonic frequency calculations using density functional theory (DFT) have been performed, to compare the theoretical results to the measured Raman spectra. Finally, electron-transfer integrals and the associated transfer rates have been calculated for all three target molecules in their crystalline form.

## Experimental Methods

### Raman Measurements

For Raman spectroscopy measurements, a WITec alpha300R confocal Raman microscope equipped with a green laser (532 nm, 20 mW) and a Zeiss Neofluar objective (40× magnification) were used. The spectra were collected through a 600 g/mm grating with the spectral center set at 1900 rel. cm^–1^ and recorded with a CCD camera (integration time: 10–20 s). The software WITec Suite FIVE was employed for cosmic ray removal and background subtraction.

### Electrode Preparation

Carbon paper (Cp, MGL370, AvCarb, thickness: 0.3 mm) discs with a diameter of 17 mm (geometric surface area of 2.27 cm^2^) were punched out with a metal cylinder. Commercially available H_2_PTCDI (TCI Chemicals, >95%), Me_2_PTCDI (TCI Chemicals, >95%), and Ph_2_PTCDI (TCI Chemicals, >95%) were purified by vacuum sublimation in a tube furnace at 380 °C for 5 h prior to evaporation. Evaporation of 250 nm thin films of the PTCDI compounds was done under vacuum (∼1 to 2 × 10^–6^ mbar) using a custom-built organic evaporation system from Vaksis R&D and Engineering, allowing precise rate control (1.2 Å s^–1^) and material heating (at 340 °C). The resulting PTCDI carbon fiber composite electrodes were used for all electrochemical and spectroscopic experiments without further treatment.

### Electrochemical Cell Assembly and Electrochemical Measurements

All electrochemical measurements were carried out in a three electrode EL-Cell (ECC-ref Cell)^21^ using a Biologic VMP3 potentiostat at room temperature. The battery half-cells are produced in an Ar-filled glovebox (UNI-lab, MBraun) with the water and oxygen contents below 0.1 ppm. Sodium metal (Na rod in paraffin oil, VWR, 99.5%) was used as counter and reference electrodes and a glass fiber disc (Ø = 18 mm, thickness 1.55 mm, EL-Cell) was used as separator. As electrolyte (Solvionic 99%) 1 M NaFSI (sodium bis(fluorosulfonyl)imide) in a 1:1 (v/v) mixture of ethylene carbonate (EC) and dimethyl carbonate (DMC) was used. All potentials are reported vs the Na/Na^+^ reference electrode. CV was performed at different scan rates from 200 to 0.05 mV s^–1^ between 3.00 and 1.00 V vs Na/Na^+^. Galvanostatic cycling with potential limitation (GCPL) was carried out between 3.00 and 1.00 V vs Na/Na^+^ at different constant currents (5, 35, 70 and 210 μA).

### Raman Spectral Analysis

The Raman spectra of H_2_PTCDI, Me_2_PTCDI, and Ph_2_PTCDI have been calculated with DFT calculations at B3LYP^22^/aug(sp)-cc-pVDZ.^23−25^ The basis sets were chosen according to the suggestions of Cheeseman and Frisch.^26^ The structures were first optimized and then the frequency calculations have been carried out using the program Gaussian 16.^27^

### Binding Energy

The binding energy over four graphite layers was calculated via SCC DFTB^28,29^ using the 3ob parameter set^30^ in conjunction with the DFT-D3 dispersion correction.^31^ The SCC DFTB calculations were performed with open-source software DFTBPLUS (version 22.1).^32^

The binding energy calculation has been done using the methodology described in a previous work,^14^ a detailed description is provided in the Supporting Information. Visualization of the structures and generation of screenshots have been carried out using VMD.^33^

### Electron Mobility

The dimer structures for H_2_PTCDI, Me_2_PTCDI, and Ph_2_PTCDI were taken from the corresponding crystal structures obtained from the CCDC database^34^ entries 187633,^35^ 1140279,^36^ and 628382,^37^ respectively. From the crystal structure, two different dimer configurations were extracted to calculate the π-stacking interaction *t*_1_ and the side-by-side interaction *t*_2_ as shown in Figure 11.

A theoretical investigation of the electron-transfer rate (interaction between the LUMOs) was performed via DFT at the B3LYP^22^/6-31+G(d)^39−42^ level using Gaussian16^27^ and incorporating the GD3BJ dispersion correction of Grimme’s dispersion with Becke-Johnson damping.^43^ The reorganization energy was determined by optimization of the single monomer and additional single-point calculations with reversed charges
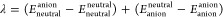
1with *E*_neutral_^anion^ being the energy of the neutral geometry with negative charge achieved by a single-point calculation and *E*_neutral_^neutral^ corresponds to the energy of the geometry optimization in the neutral state. *E*_anion_^anion^ and *E*_anion_^neutral^ are the energies of the optimized anionic state and the single-point calculation of the anionic structure in the neutral state, respectively.

The electron-transfer rates were calculated using the Marcus–Hush equation^44−46^
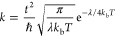
2where *t* is the transfer integral between neighboring molecules in the organic crystal, *T* is the temperature, *k*_b_ and λ are the Boltzmann constant and the reorganization energy, respectively. The generalized transfer integral *t* (oftentimes also referred to as *J*_eff_) is obtained from the results of the DFT calculations as
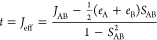
3with *J*_AB_ and *S*_AB_ being the respective transfer and overlap integrals and *e*_A_ and *e*_B_ are the associated site energies. The calculations of *J*_eff_ have been carried out using the open-source program CATNIP (version 1.9).^47−49^

## Results and Discussion

**Figure 1:**
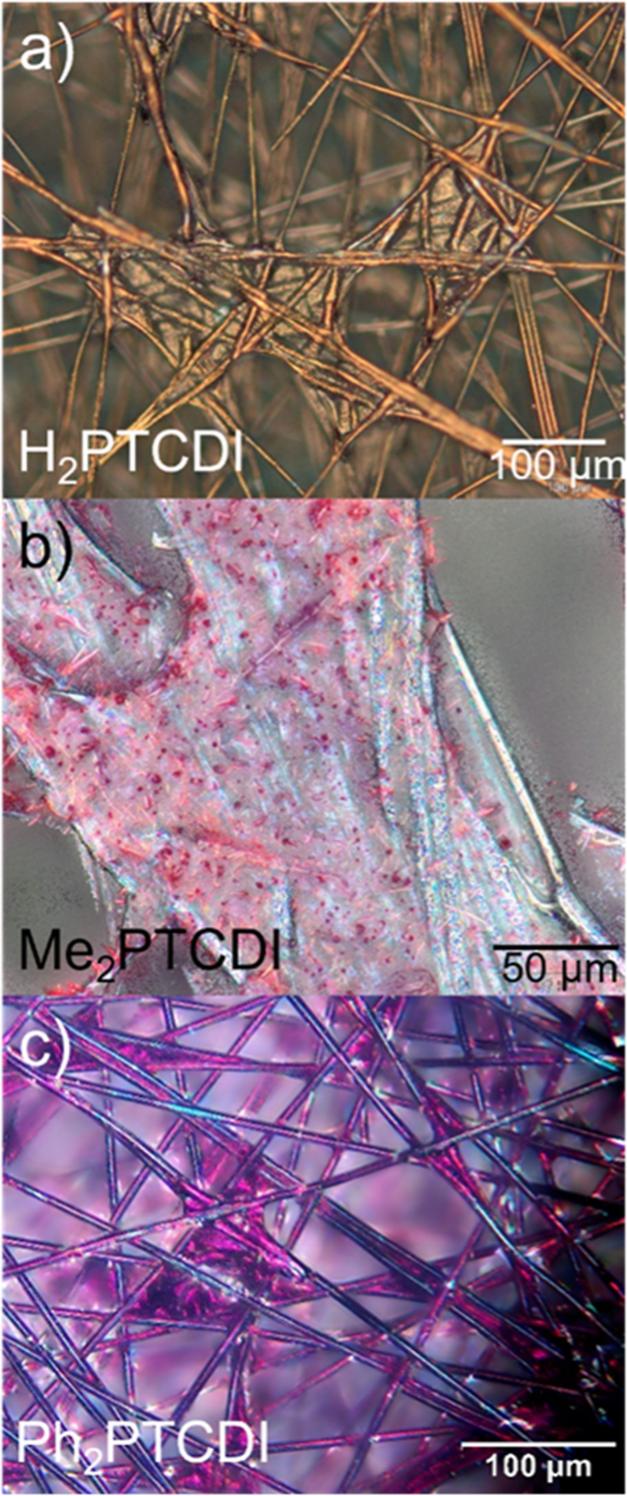
Optical microscope images of the three different PTCDI carbon fiber composite electrodes: (a) H_2_PTCDI, (b) Me_2_PTCDI, and (c) Ph_2_PTCDI with a magnification of 20×, 50×, and 20×, respectively.

**Figure 2:**
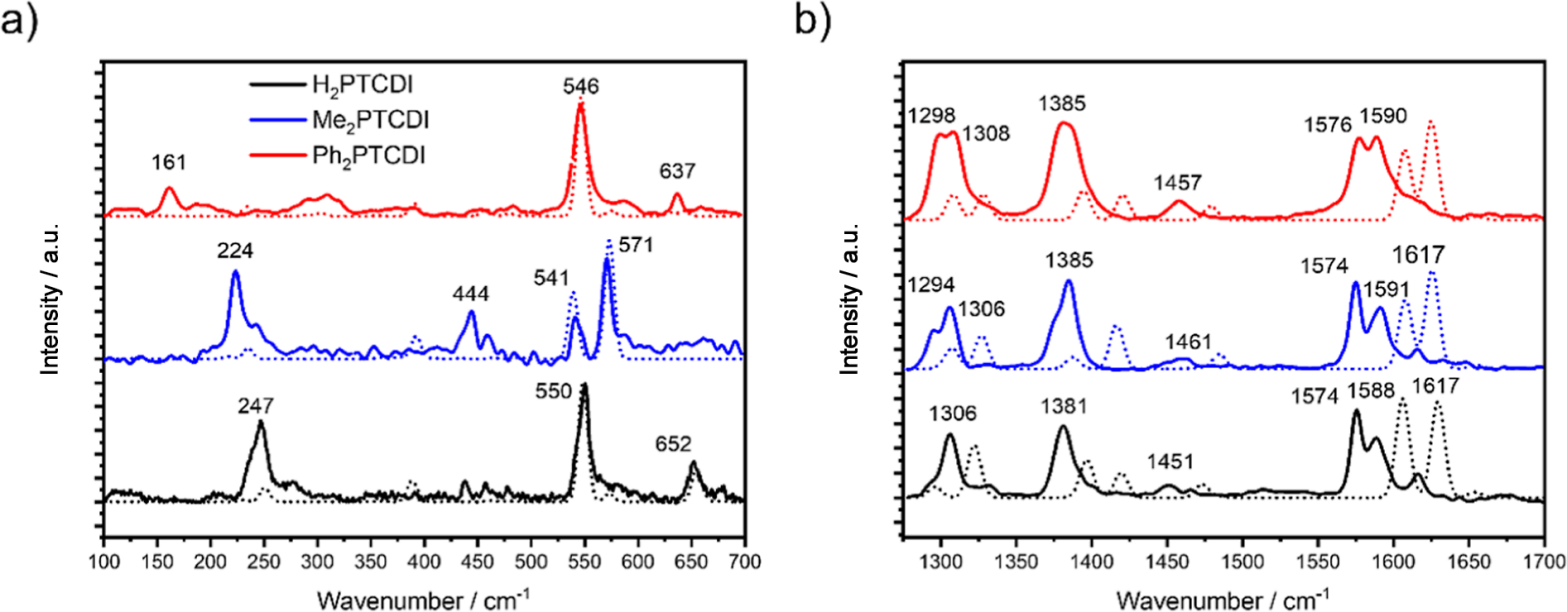
Raman spectra of H_2_PTCDI (black), Me_2_PTCDI (blue), and Ph_2_PTCDI (red) cp composite electrodes (laser wavelength 532 nm). The calculated spectra are given as dashed lines. (a) Spectra for the wavenumber region of 100–700 cm^–1^. (b) Spectra for the wavenumber region of 1275–1700 cm^–1^. Peak wavenumbers are indicated above the peaks. The peak intensities are normalized to the highest intensity peak: peak located at 550 cm^–1^ for (a) and peak located at 1380 cm^–1^ for (b) [for the calculated spectra, the peak intensities are normalized to the peak at 1625 cm^–1^ for (b)]. The assignment of the bands is given in Table S1.

The carbon fiber substrates are coated with a 250 nm thick PTCDI film via a thermal evaporation process. For spectroscopic characterization of the PTCDI films, Raman spectroscopy is used. The Raman spectra of the carbon fiber substrate are characterized by the typical D, G, and 2D bands for graphitic materials (see Figure S2). The ratio between the intensities of G and D bands, which is produced by defects and disorder in the graphite lattice, is 0.06, which correlates to a nearly defect-free material.^20,50^ The 2D band is split into portions of 2D_1_ and 2D_2_, indicating a three-dimensional ABAB stacking of the single layers.^51^ In Figure 1, optical images of the three different PTCDI composite electrodes are shown. The 20× magnification of the H_2_PTCDI and Ph_2_PTCDI carbon fiber composite electrode images (Figure 1a,c) reveal the substrate as an interconnected network of carbon fibers coated with a homogeneous PTCDI film wrapping around the carbon fibers, forming for H_2_PTCDI an almost closed hull. In the magnified image (50×) of Me_2_PTCDI (Figure 1b), the PTCDI molecules are adsorbed as red, crystalline needles on top of the carbon fiber. The Raman spectra, shown in Figure 2, of all three PTCDI composite electrodes are dominated by the fundamental frequencies of the perylene core (Figure 2b).^52^ The peaks at 1300, 1380, and 1575 cm^–1^ can be assigned to the in-plane ring stretch, (C–H)-bend, and (C=C) stretch vibrations of the core molecule.^53^ Interestingly, the Raman bands in this region are rather broad or even split into peak doublets. This is due to the long-range order of the PTCDI films, presumably produced by intermolecular interactions like π–π stacking and hydrogen bonding between the single molecules in the film.^53−55^ In contrast to this behavior, surface-enhanced-resonance-Raman-spectroscopy (SERS) measurements of PTCDI films on silver islands show only single peaks in this region, since both the electromagnetic and chemical effects are short-range in the SERS experiment.^53^ All three PTCDI molecules show a band below 250 cm^–1^ (247 cm^–1^ for H_2_PTCDI, 224 cm^–1^ for Me_2_PTCDI, and 161 cm^–1^ for Ph_2_PTCDI). Assuming that these peaks correspond to the same vibrational mode,^53^ the frequency values of these peaks could be affected by the mass of the different residuals on the nitrogen atom. An increase of mass would decrease the frequency of the vibration, resulting in a downshift of the peak wavenumber. As it can be seen in Figure 2a, this is corroborated by the lowest wavenumber for the Ph_2_PTCDI vibrational mode at 161 cm^–1^. Therefore, the bands can be assigned to a bending vibration of the C–N–C group, allowing qualitatively to distinguish the three different PTCDI molecules.^53,56^ Qualitatively, the calculated Raman spectra are in good agreement with the experimental data as all the fundamental Raman bands are resolved. However, the calculated Raman wavenumbers, especially in Figure 2b, are blue-shifted by a factor of around 1.02. On the one hand, DFT methods are known to slightly overestimate vibrational frequencies,^57^ and on the other hand, the influence of the carbon fiber substrate is unaccounted for in the theoretical calculations.

**Figure 3:**
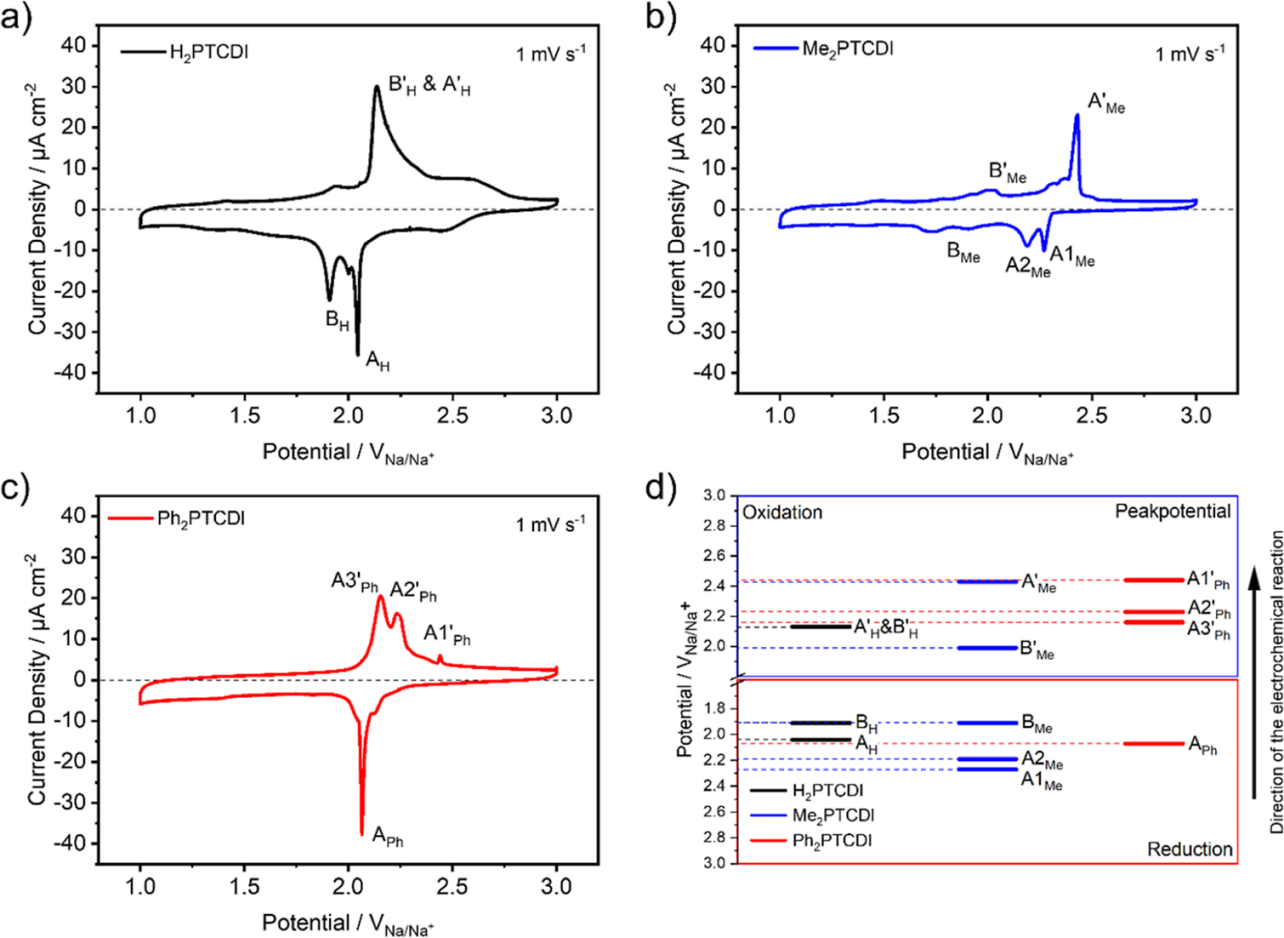
CV measurements of a 250 nm thick film of (a) H_2_PTCDI, (b) Me_2_PTCDI, and (c) Ph_2_PTCDI on carbon fiber with a scan rate of 1 mV s^–1^ in a 1 M NaFSI/EC/DMC [1:1 (v/v) mixture] electrolyte. (d) Peak potentials of the reduction peaks are denoted as A and B and back-oxidation peaks are denoted as A′ and B′ for the three different PTCDI molecules.

To characterize the changes in electrochemical performance, arising from exchanging the residual on the nitrogen atom, three different PTCDI composite electrodes with 250 nm thick films are further investigated as battery half-cells with an organic electrolyte containing Na ions and sodium metal counter and reference electrodes. Such PTCDI carbon fiber composite electrodes in a Na-ion battery half-cell are defined by a three-component system composed of the liquid electrolyte, the solid PTCDI film, and the solid carbon fiber substrate. Therefore, the PTCDI films are sandwiched between a solid/solid interface with the carbon fiber substrate and a liquid/solid interface with the electrolyte. As the PTCDI films are conducting for both, the Na ions and the electrons, the material is considered as a mixed electron–ion conductor. The solid/solid interface of the film and the carbon fiber is acting as blocking boundary for the Na ions as no intercalation of Na ions into the carbon fiber substrate takes place (compare Figure S3). Generally, the PTCDI molecules are known to undergo a reversible two-electron redox reaction in the potential range from 1 to 3 V versus Na/Na^+^ (see Figure S1).^18,58,59^ Therefore, two reduction peaks and two back-oxidation peaks are expected in the CV, corresponding to the reduction of the neutral PTCDI molecules to the radical anion, the reduction of the radical anion to the dianion, and the corresponding back-oxidation reactions. Figure 3 shows CV measurements with a scan rate of 1 mV s^–1^ for H_2_PTCDI, Me_2_PTCDI and Ph_2_PTCDI, respectively. Starting from 3 V going in the reductive direction, the H_2_PTCDI composite electrode produces a current plateau beginning at around 2.5 V cumulating in two sharp reduction peaks at 2.04 V (Figure 3a, A_H_) and 1.91 V (Figure 3a, B_H_). The back-oxidation is characterized by a tailing back-oxidation peak at 2.13 V (Figure 3a, B_H_′, and A_H_′) followed by a current plateau until about 2.60 V. Small post- and prepeaks can be observed after and before the reduction peak A_H_ and back-oxidation peak B_H_′ and A_H_′, respectively. The Me_2_PTCDI composite electrode shows a sharp peak couple at 2.27 V (Figure 3b, A1_Me_) and at 2.19 V (Figure 3b, A2_Me_) followed by an undulating broad peak at 1.91 V (Figure 3b, B_Me_).

The back-oxidation reaction shows a similar broad peak at 1.99 V (Figure 3b, B_Me_′) and a rather sharp second back-oxidation peak at 2.43 V (Figure 3b, A_Me_′). The Ph_2_PTCDI composite electrode shows, differently to the two other materials, only one very sharp reduction peak at 2.07 V (Figure 3c, A_Ph_) while the back-oxidation is split into three peaks. A peak doublet at 2.16 V (Figure 3c, A3_Ph_′) and 2.23 V (Figure 3c, A2_Ph_′) is followed by a small peak at 2.44 V (Figure 3c, A1_Ph_′). Figure 3d illustrates the peak potential differences of the redox reactions for all three electrodes. In principle, the first reduction peak should originate from the reduction of the neutral PTCDI molecule to the radical anion. This reduction occurs at the highest potential for Me_2_PTCDI (Figure 3d, A1_Me_) followed by Ph_2_PTCDI and H_2_PTCDI, which have approximately the same reduction potential, with a potential difference of 230 mV compared to Me_2_PTCDI. In the second reduction step, the dianion is formed from the intermediate radical anion. This reduction is only resolved as a separate peak for H_2_PTCDI (Figure 3a, B_H_). For Ph_2_PTCDI, the two-step reduction is merged into one peak. For both Me_2_PTCDI, the peak A2_Me_ or the broad peak B_Me_ could be the manifestation of the second reduction step.

The main back-oxidation peaks B_H_′ and A_H_′ for H_2_PTCDI, A3_Ph_′ for Ph_2_PTCDI, and A_Me_′ for Me_2_PTCDI show a similar behavior as the peaks for Ph_2_PTCDI and H_2_PTCDI appear at similar potentials while the peak for Me_2_PTCDI is shifted 300 mV in the oxidative direction. However, the CV response of the three different PTCDI composite electrodes with peak splitting, peak merging, and overall unsymmetrical current response clearly does not show a simple reversible, two-electron redox reaction of the PTCDI molecules as usually described in theory. In contrast, it highlights the influence of the film structure and geometry of the three different PTCDI carbon fiber composite electrodes. To further investigate the electrochemical characteristics of these systems, the battery half-cells are cycled with several different scan rates ranging from 200 to 0.05 mV s^–1^ (comp. Figures S4–S6). The dependence of peak current *I*_p_ with scan rate ν gives important insights into the process which limits the reaction current.^60^

In Table 1, the resulting slopes of a linear fit for the logarithm of peak currents versus the logarithm of scan rate plots (Figures S7–S9) are recorded for the different peaks of the H_2_-, Me_2_-, and Ph_2_PTCDI composite electrodes.

**Table 1:**
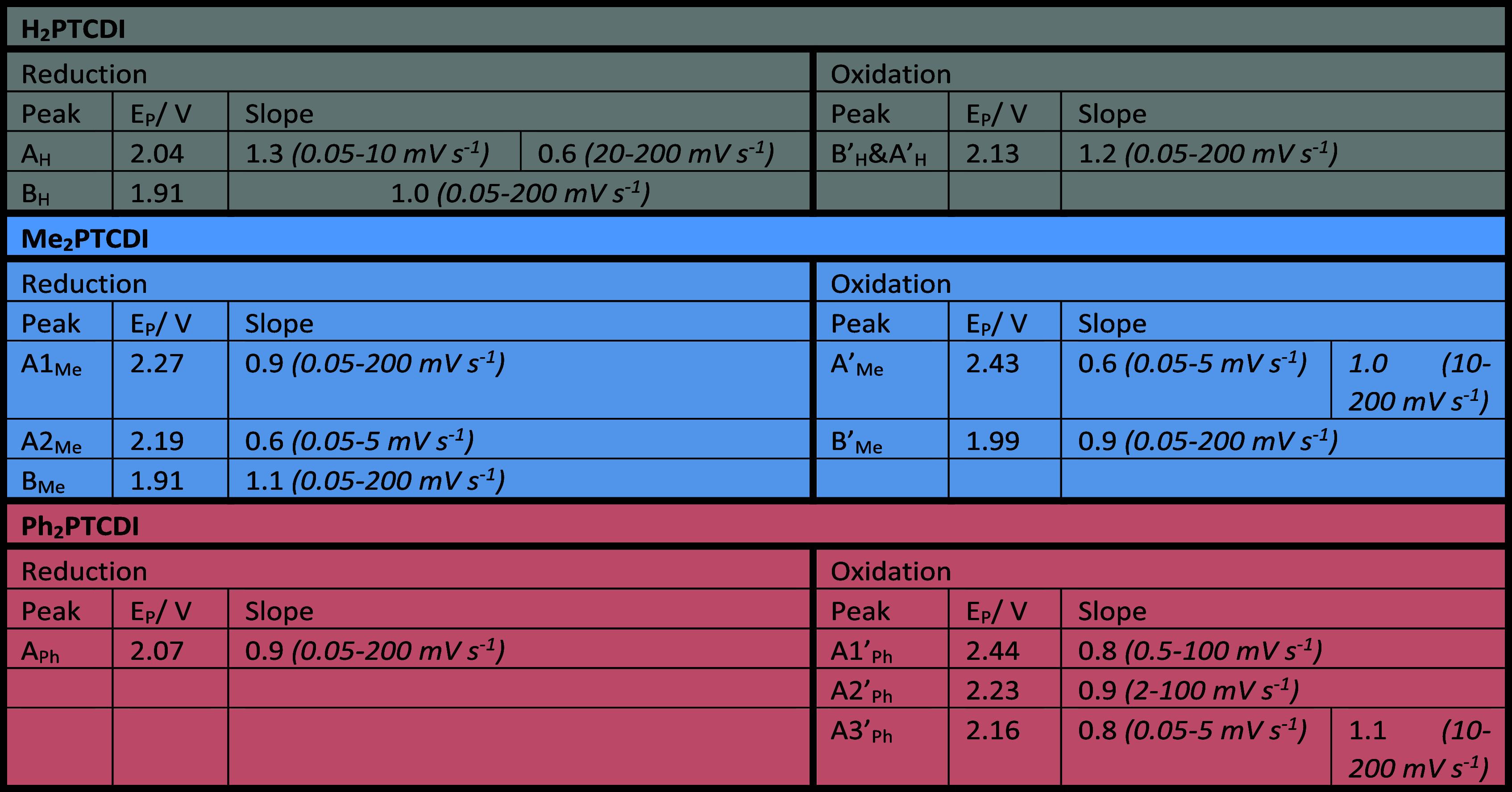
Reduction and Oxidation Peak Potentials *E*_P_ for H_2_-, Me_2_-, and Ph_2_PTCDI^a^

a Slopes for the linear fit of the logarithm of the peak currents versus the logarithm of the scan rate plots. The values of the peak currents *I*_P_ are the absolute values of the respective CV measurements, having the capacitive current for the pure carbon paper substrate without active material subtracted.

A slope of 1 indicates that the peak current is proportional to the scan rate while a slope of 0.5 relates the dependence of the peak current to the square root of the scan rate. Most of the peak currents are proportional to the scan rate, which is characteristic for a capacitive behavior.^60^ This is typical for a surface-confined system where the diffusion of neither counterions nor electrons through the film is rate-limiting.^61^ For the H_2_PTCDI carbon fiber composite system, this behavior deviates in the fast scan rate range (20–200 mV s^–1^) for the first reduction peak A. In this region, the peak current is proportional to the square root of the scan rate and a diffusion process becomes rate-limiting. Interestingly, in the Me_2_PTCDI system, the back-oxidation peak A′ switches its behavior differently, from a capacitive response in the fast scan rate range (10–200 mV s^–1^) to a diffusive response in the slow scan rate range (0.05–5 mV s^–1^). At first, this is counterintuitive, but it becomes reasonable upon investigation of the reductive peaks A1_Me_ and A2_Me_. The first reduction peak A1_Me_ is proportional to the scan rate over the whole scan rate range (0.05–200 mV s^–1^) while the second reduction peak A2_Me_ has a dependence on the square root of the scan rate and is only visible in the slow scan rate range (0.05–5 mV s^–1^, Figure S5). Hence, the back-oxidation peak A_Me_′ can be interpreted as a superposition of two back-oxidation reactions correlating to the reduction reactions occurring at the peaks A1_Me_ and A2_Me_. In the fast scan rate range, the reduction reaction at peak A1_Me_ dominates, therefore the back-oxidation peak A_Me_′ has a capacitive response, while in the slow scan rate range, the reduction reaction at peak A2_Me_ dominates and the response of the back-oxidation peak switches to a diffusive behavior. The charge stored increases with the decreasing scan rate for both the reduction peak A2_Me_ and back-oxidation peak A_Me_′, whereas the charge stored at the reduction peak A1_Me_ stays constant with the changing scan rate (see Figure S10). This behavior could be rationalized the following way: The reduction reaction occurring at the reduction peak A1_Me_ corresponds to a part of the Me_2_PTCDI film adjacent to the carbon fiber substrate. The further reduction of the bulk is mediated through an exchange reaction of this adjacent layer with the rest of the film. It appears that the exchange reaction rate is limited through the slow electron transfer between the molecule layers, which results in a diffusional behavior of the reduction peak A2_Me_.^62^ The appearance of a single peak at the back-oxidation reaction is caused by the mediation of the bulk reaction through the back-oxidation of the adjacent Me_2_PTCDI layer to the carbon fiber substrate. The back-oxidation reaction can only start if the adjacent layer gets oxidized first (even though the potential at which the bulk reaction occurs is already reached) resulting in a very sharp back-oxidation peak. Moreover, this behavior is evident in the CVs of different film thicknesses, wherein the current of the reduction peak A2 is dependent on the film thickness, nearly vanishing for a 50 nm thick film (Figure S11). Next to the change of the peak current, the change of peak potential with the scan rate is an important characteristic giving information about the reversibility and kinetic limitations of a reaction.^63^ For surface-confined systems, the peak potentials of the reduction and oxidation reactions are the same, hence the peak potential difference Δ*E*_R/O_ is zero. The peak potentials start to vary from their equilibrium potentials when electron-transfer kinetics become limiting.^61,64^ The peak potentials for the three PTCDI composite electrodes remain almost constant over a wide range of scan rates (0.05–20 mV s^–1^) and only shift notably to more reductive potentials for the reduction reactions and to more oxidative potentials for the oxidation reactions at the fast scan rate range (50–200 mV s^–1^) (Figures S12–S14). However, different to the ideal surface-confined system a peak potential difference Δ*E*_R/O_ is always prevalent e.g., the peak potential difference at the slowest scan rate of 0.05 mV s^–1^, between the first reduction peak A1_Me_ and the back-oxidation peak A_Me_′, is 120 mV for the Me_2_PTCDI composite electrode system. Furthermore, the peak shape is hardly ever symmetric with a full width at half-maximum (fwhm) δ of 90.6/N mV, given an ideal surface-confined system.^65^ Most of the peaks are much sharper than the theoretical value.

**Figure 4:**
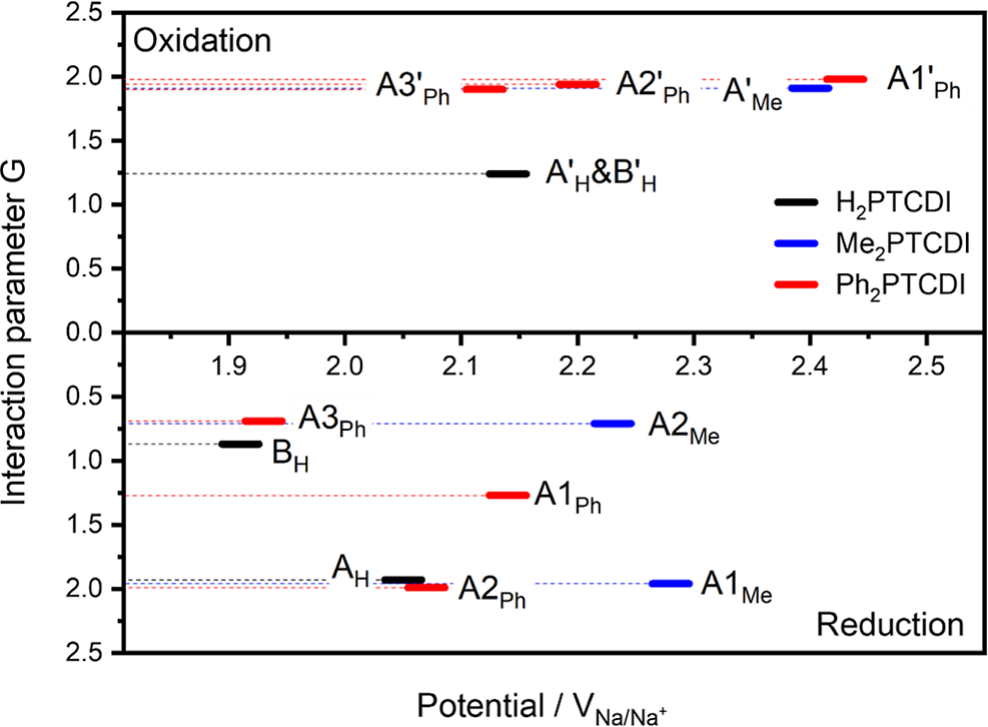
Interaction parameters G for H_2_PTCDI (black), Me_2_PTCDI (blue), and Ph_2_PTCDI (red) for the reduction peaks in the lower half of the plot and for the oxidation peaks in the upper half of the plot. The values of *G* are positive for all peaks. The abscissas values indicate the respective redox potentials.

According to the theoretical work of Laviron,^66,67^ the discrepancy between the ideal fwhm of a surface-confined system and the fwhm values for PTCDI can be explained by interactions between the confined molecules. For a reversible reaction of a surface-confined electrode, Tokuda et al.^68^ gave a theoretical equation which links the fwhm δ with an interaction parameter *G*.
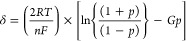
4With
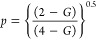
5*R* is the ideal gas constant, *F* is the Faraday constant, *T* is the temperature, and *n* is the number of transferred electrons in the reaction step. For attractive interactions, the peaks become sharper and consequently the fwhm δ becomes less than 90.6/N mV, resulting in a positive interaction parameter *G*. Likewise, for repulsive interactions, the peaks are broadened, and the interaction parameter *G* has a negative value. In Figure 4, the respective values for the interaction parameter *G* for each redox peak are plotted versus their peak potentials. The first reduction peaks (A_H_ for H_2_PTCDI, A1_Me_ for Me_2_PTCDi, and A2_Ph_ for Ph_2_PTCDI) are all very sharp and have an interaction parameter of nearly 2. In Laviron’s theory, the limiting positive interaction parameter value is 2.^67^

**Figure 5:**
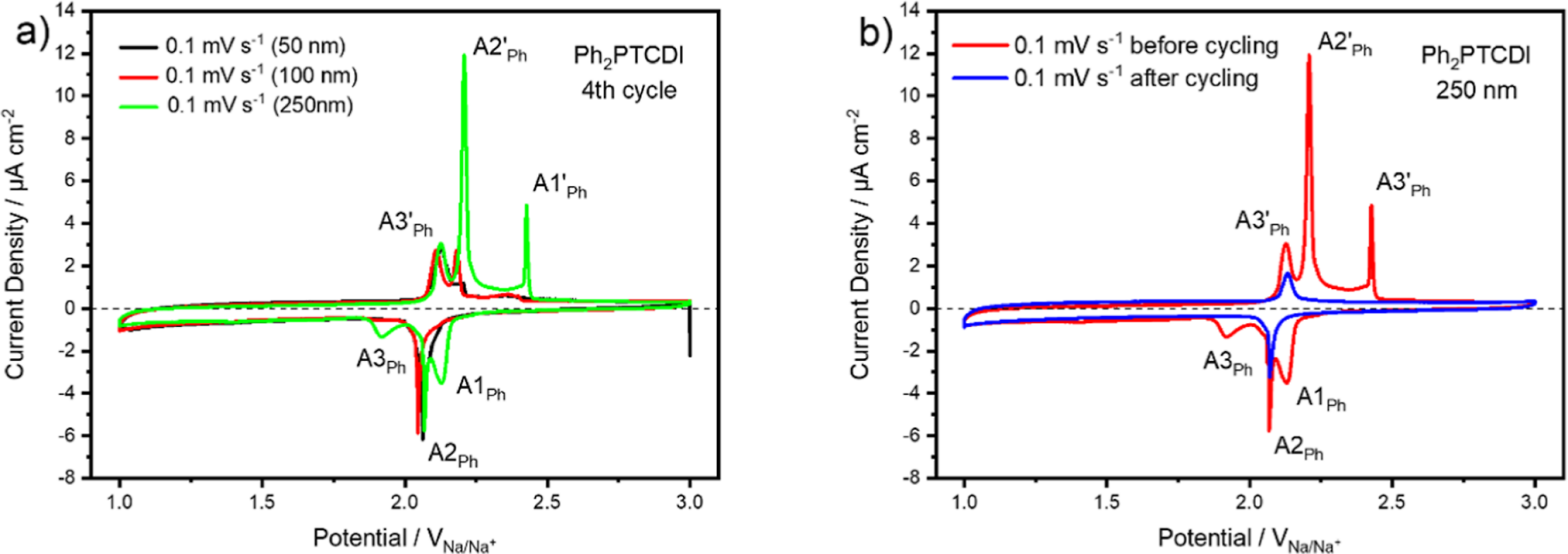
CV data of a Ph_2_PTCDI carbon fiber composite electrode. (a) Comparison of the CV data for 50 (black), 100 (red), and 250 (green) nm thick Ph_2_PTCDI films at a scan rate of 0.1 mV s^–1^. The fourth cycle is depicted. (b) Comparison of the CV for a 250 nm thick Ph_2_PTCDI film before (red) and after (blue) doing 300 GCPL cycles at a scan rate of 0.1 mV s^–1^.

**Figure 6:**
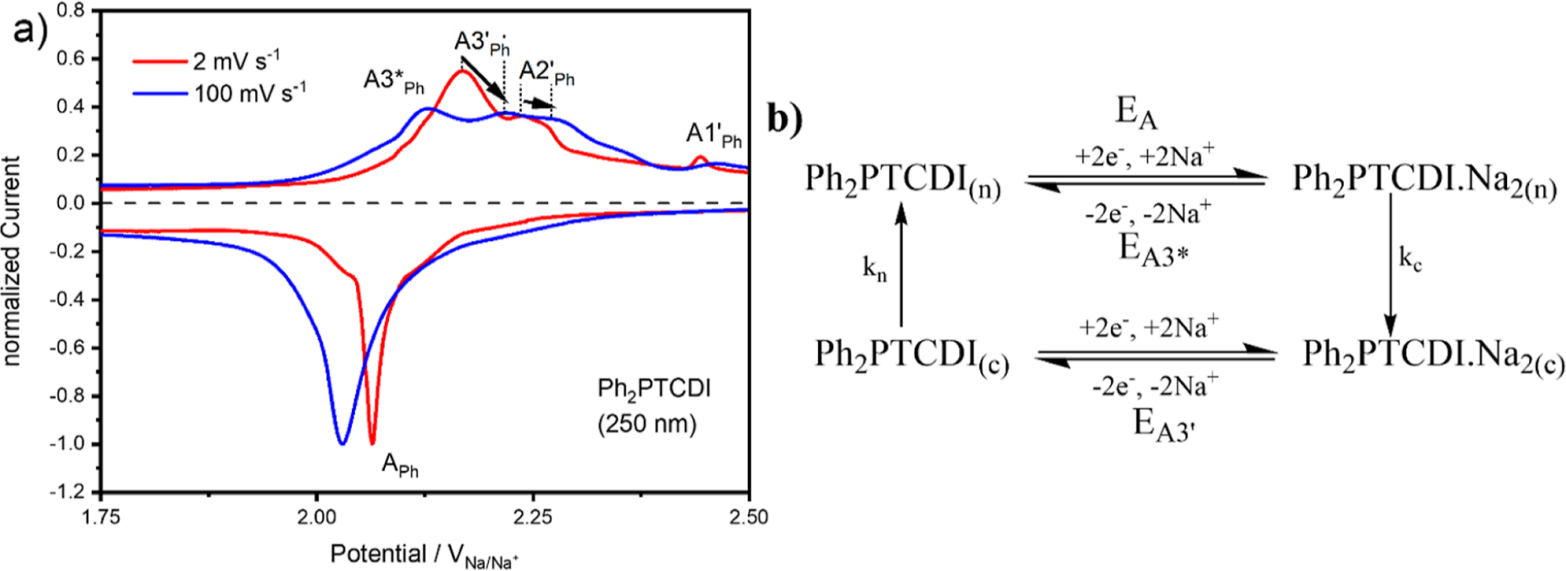
(a) CV of a 250 nm thick Ph_2_PTCDI carbon fiber composite electrode with scan rates of 2 (red) and 100 (blue) mV s^–1^ from 2.5 to 1.75 V. The current is normalized to the peak current of peak A. The new peak appearing at the scan rate of 100 mV s^–1^ is denoted as A3_Ph_*. The arrows indicate the peak potential shift induced by kinetic limitations. (b) Proposed square scheme for the redox reaction of arrangement 1. (*n*) is the neutral and (c) the charged configuration of arrangement 1. *k*_c_ and *k*_*n*_ are the rate constants for the conversion reaction from the neutral to the charged configuration and vice versa. E_A_, E_A3*_, and E_A3′_ are the potentials of the respective reduction/back-oxidation reactions.

For values above/equal 2 the theory collapses as the peaks would become infinitely sharp and infinitely high (see eqs 4 and 5: for *G* = 2; *p* becomes zero and therefore the fwhm δ becomes zero). The limit of extremely sharp peaks often indicates a phase transition.^65,69^ Likewise, the back-oxidation peaks A_Me_′ for Me_2_PTCDI and A3_Ph_′, A2_Ph_′, and A1_Ph_′ for Ph_2_PTCDI have interaction parameters close to 2. The back-oxidation peak (B_H_′ and A_H_′) for H_2_PTCDI is not suitable for this analysis as it has a tailing peak, and it might be that the two back-oxidation reactions are not resolved in the CV measurements as single peaks (due to their close peak potentials), or the peak represents a one-step two-electron oxidation reaction. The reduction peak A2_Me_ for Me_2_PTCDI is limited by a diffusion-controlled process, which influences the fwhm. The second reduction peak B_H_ for H_2_PTCDI has an interaction parameter of 0.87. Therefore, it can be concluded that the radical anions form a film of H_2_PTCDI molecules with weaker attractive interactions in comparison to the neutral H_2_PTCDI molecules. The two additional reduction peaks A1_Ph_ and A3_Ph_ for Ph_2_PTCDI only appear at the slowest scan rates. In Figure 6a, the dependency of the CV response upon film thickness at a scan rate of 0.1 mV s^–1^ is depicted. For the 250 nm thick film (Figure 5a, green curve) two new reduction peaks appear, the reduction peak A1, with a potential of 2.14 V, and the reduction peak A3, with a potential of 1.93 V. The reduction peak A2_Ph_ is at a potential of 2.07 V, which is at the same potential as the reduction peak A_Ph_ in Figure 3c. The three back-oxidation peaks A3_Ph_′, A2_Ph_′, and A1_Ph_′ are at potentials of 2.12, 2.20, and 2.43 V and are slightly shifted to less anodic potentials when compared to the back-oxidation peaks in Figure 3c.

**Figure 7:**
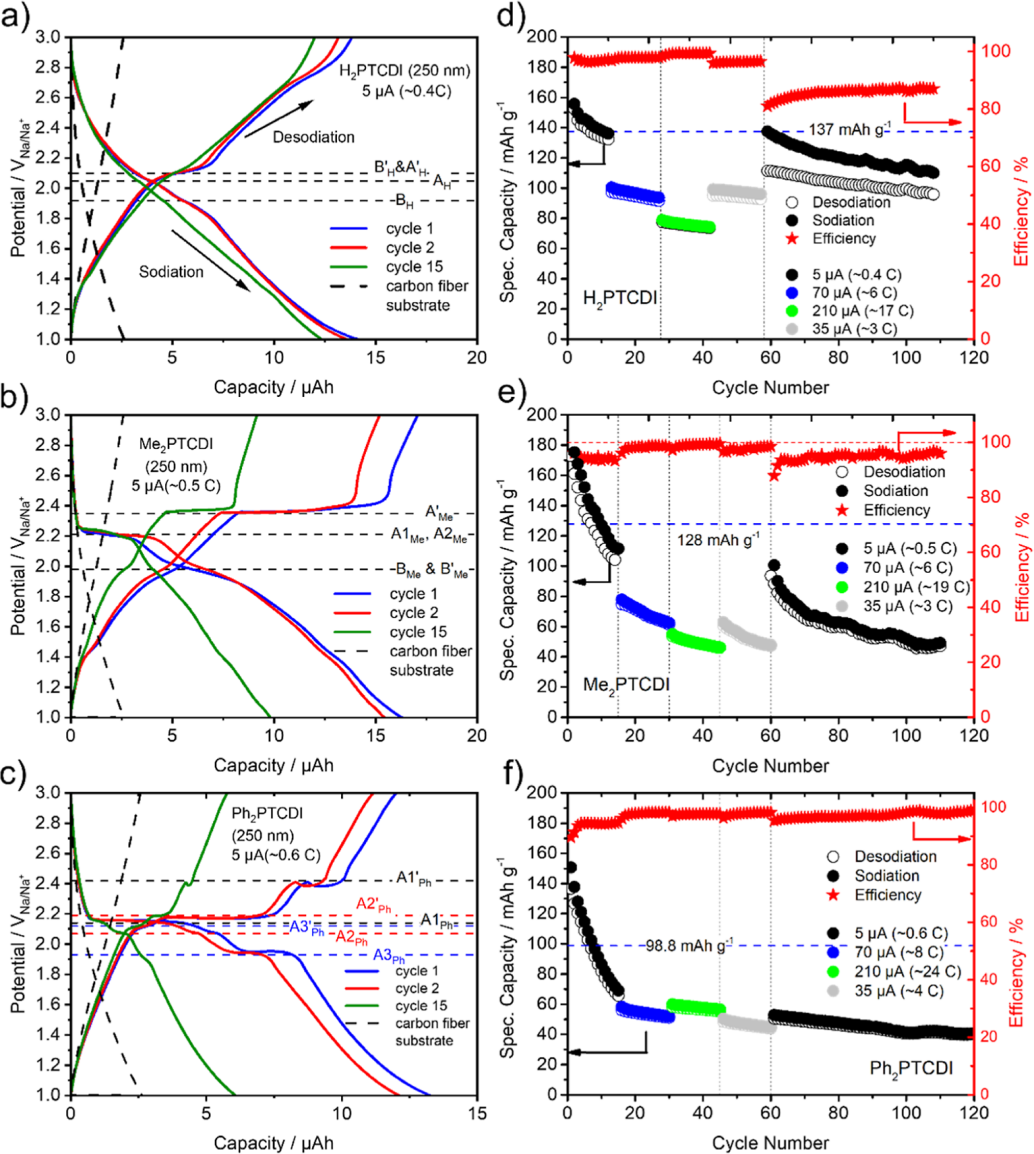
(a–c) Galvanostatic charge/discharge performances of the PTCDI carbon fiber composite electrode (250 nm) for the first 15 cycles with an applied constant current of 5 μA. The charge/discharge profile of the carbon fiber substrate without active material is given as dashed lines. (d–f) Specific capacity (charge, open circles; discharge, closed circles) of the PTCDI films with different applied constant currents of 5 μA (black circles), 70 μA (blue circles), 210 μA (green circles), and 35 μA (gray circles) and the corresponding calculated efficiencies versus cycle number.

However, the back-oxidation peaks A2_Ph_′ and A1_Ph_′ become very sharp. The 100 nm (Figure 6a, red curve) and 50 nm (Figure 5a, black curve) thick films have only one reduction peak located at the potential of A2_Ph_. The back-oxidation for these two film thicknesses has a back-oxidation peak located at the potential of A3_Ph_′. At the potential of the back-oxidation peak A2_Ph_′, the 100 nm thick film shows a peak whereas the 50 nm thick film is characterized by a current plateau. At the region of the back-oxidation peak A1_Ph_′, only a small broad peak is detectable. Interestingly, the reduction peak A2_Ph_ and the back-oxidation peak A3_Ph_′ have the same height for all three film thicknesses. In Figure 5b, the CV responses at a scan rate of 0.1 mV s^–1^, of a 250 nm Ph_2_PTCDI electrode before and after 300 cycles of GCPL measurements, are compared. The reduction and back-oxidation peaks A1_Ph_, A3_Ph_, A2_Ph_′, and A1_Ph_′ vanish after 300 GCPL cycles, whereas the reduction peak A2_Ph_ and the back-oxidation peak A3_Ph_′ remain at their potentials but are reduced in magnitude. This behavior suggests that the Ph_2_PTCDI molecules are adsorbed in two different arrangements. The first arrangement gets reduced at the potential of peak A2_Ph_ and back-oxidized at the potential of peak A3_Ph_′. The second arrangement gets reduced in two steps, at the peak potentials of the peaks A1_Ph_ and A3_Ph_ and back-oxidized at the potentials of peak A2_Ph_′ and A3_Ph_′. Hence, the peaks A2_Ph_ and A3_Ph_′ correspond to arrangement 1 while the peaks A1_Ph_, A3_Ph_, A2_Ph_′, and A1_Ph_′ correspond to arrangement 2. It also appears that the Ph_2_PTCDI molecules are adsorbed in arrangement 1 for small coverages and begin to adsorb in arrangement 2 when a certain coverage of Ph_2_PTCDI molecules on top of the carbon fiber substrate is reached. This is also visible in optical images of Ph_2_PTCDI electrodes with different film thicknesses as there is a color change from violet for small coverages to golden red for bigger coverages (Figure S15). Furthermore, the active material in arrangement 2 seems to get inactivated more rapidly upon GCPL experiments (Figures 5b and 7c). Additionally, the redox reaction corresponding to arrangement 2 seems to be kinetically hindered as the redox reaction only proceeds at the slow scan rates (0.1–0.05 mV s^–1^). Such a dependence on film thickness was not present for the H_2_PTCDI films (Figure S16). Another interesting phenomenon is the apparent shift in the cathodic direction of the peak potential of A3_Ph_′ with an increasing scan rate (Figure S13, open blue squares). In Figure 6a, the CV of a 250 nm thick Ph_2_PTCDI carbon fiber composite electrode with scan rates of 2 (red) and 100 (blue) mV s^–1^ from 2.5 to 1.75 V is illustrated. The current is normalized to the peak currents of peak A_Ph_. At the scan rate of 100 mV s^–1^, a new back-oxidation peak A3_Ph_* appears at the potential of 2.13 V while the back-oxidation peaks A3_Ph_′ and A2_Ph_′ are shifted to more anodic potentials due to kinetic limitations (indicated with arrows in Figure 6a). This new peak can be explained via the square scheme shown in Figure 6b. The Ph_2_PTCDI molecules in arrangement 1 (peaks A_Ph_ and A3_Ph_′) are initially in a configuration corresponding to their neutral state [indicated with (*n*) in the square scheme].

The molecules get reduced at the potential E_A_ (peak A_Ph_ in Figure 6a) and upon reduction the now negatively charged molecules transform their structure into a configuration appropriate for their charged state [indicated with (c) in the square scheme]. The speed of the transformation is controlled by the rate constant *k*_c_. At fast scan rates (e.g., 100 mV s^–1^), the molecules are not completely transformed into their charge appropriate configuration upon reaching the potential of the back-oxidation reaction E_A3*_. Hence, a new peak A3_Ph_* appears in the CV, corresponding to the back-oxidation of the reduced Ph_2_PTCDI molecules in the neutral configuration.

To assess the long-term stability of the PTCDI composite electrodes, GCPL measurements are performed. Figure 7a–c shows the GCPL data in the potential range from 1 to 3 V versus Na/Na^+^, with an applied constant current of 5 μA for the three different systems. Generally, the galvanostatic cycling response (Figure 7a–c) of the three PTCDI films agrees with the CV measurements as the potential plateaus coincide with the CV peaks (peak potentials are denoted as horizontal dashed lines in Figure 7a–c). However, while the H_2_PTCDI composite electrode has sloping plateau-like regions around the peak potentials, the Me_2_PTCDI and Ph_2_PTCDI composite electrodes show almost horizontal plateaus at the CV peak potentials. In the first cycle, the sodiation/desodiation capacities for H_2_PTCDI, Me_2_PTCDI, and Ph_2_PTCDI are 14.1/13.8, 16.3/17.0, and 13.2/12.0 μA h, respectively. This corresponds to specific gravimetric capacities of 155/152 mAh g^–1^ for H_2_PTCDI, 185/193 mAh g^–1^ for Me_2_PTCDI, and 150/136 mAh g^–1^ for Ph_2_PTCDI. These values are higher than their corresponding theoretical capacities of 137, 128, and 98.8 mAh g^–1^, respectively. Some of the additional capacity can be attributed to the carbon fiber substrate as it has itself a capacity contribution of 2.6 μA h for an applied constant current of 5 μA (see the dashed line in Figure 7a–c). However, especially for the Me_2_PTCDI and Ph_2_PTCDI systems, this contribution cannot fully explain the measured overcapacity. Some contribution may result from side reactions that occur at the first cycle or because the density of the PTCDI films is higher than in their respective single crystals (which was used for the mass calculation). After 15 cycles, the sodiation/desodiation capacities for H_2_PTCDI, Me_2_PTCDI, and Ph_2_PTCDI decrease to 12.3/12, 9.8/9.1, and 6/5.7 μA h, respectively. This correlates to an active material loss of 13% for H_2_PTCDI, 40% for Me_2_PTCDI, and 55% for Ph_2_PTCDI (under the assumption that the other contributions to the capacity remain constant). At the 110th cycle, the H_2_PTCDI electrode lost 30%, the Me_2_PTCDI electrode 74%, and the Ph_2_PTCDI electrode 73% of their initial capacity. In Figure 7d–f, the obtained values of the specific gravimetric capacity and the corresponding Coulombic efficiency over 110 cycles, with different constant currents [see Figure 7d–f; 5 μA (black circles), 70 μA (blue circles), 210 μA (green circles), and 35 μA (gray circles)] are depicted. The Coulombic efficiencies for the elevated constant currents (35–210 μA) are in the range of 96–100% for all three PTCDI composite electrodes, showing a good reversibility at fast charging rates. However, the H_2_PTCDI composite electrode (and to some extent the Me_2_PTCDI composite electrode) shows a severe drop in Coulombic efficiency when returning to the low constant current of 5 μA for 50 cycles, with a Coulombic efficiency of 81% at the first and 87% at the 50th cycle. This drop in efficiency could be a result of irreversible side reactions with the electrolyte as it is also visible for the pure carbon fiber substrate, around 1 V during sodiation (see Figure S17). The contribution of these side reactions becomes more pronounced at prolonged cycling as some of the capacity of the active material is lost while the capacity of the side reactions remains constant. The same is observed for cycling at low constant currents as the side reactions have more time to progress. Interestingly, the Ph_2_PTCDI film seems to inhibit these side reactions. Some interesting properties are noticeable when comparing the specific gravimetric capacities with the applied constant currents for the three different PTCDI composite electrodes. H_2_PTCDI and Me_2_PTCDI retain 58 and 49% of their capacity (the capacities of the 15th cycles with a constant current of 5 μA are used as reference points) when a constant current of 210 μA is applied, corresponding to 17 C for H_2_PTCDI (charging time of 3.5 min) and 19 C for Me_2_PTCDI (charging time of 3.2 min) whereas Ph_2_PTCDI retains 88% of its capacity when applying a constant current of 210 μA (24 C or a charging time of 2.5 min). This high retention at very fast charging rates indicates a fast sodiation/desodiation mechanism.

Furthermore, the gravimetric capacity of the Ph_2_PTCDI electrode increases from 58/56 mAh g^–1^ for an applied current of 70 μA (Figure 7f, blue) to 60/58 mAh g^–1^ for an applied current of 210 μA (Figure 7f, green). An increase of the specific capacity with increasing applied current is counterintuitive. However, this behavior may be rationalized by the square scheme shown in Figure 6b as the redox pathway for shorter charging times becomes different. In summary, while all three composite electrodes show high initial specific gravimetric capacities, the cycle stability of the H_2_PTCDI composite electrode is superior.

**Figure 8:**
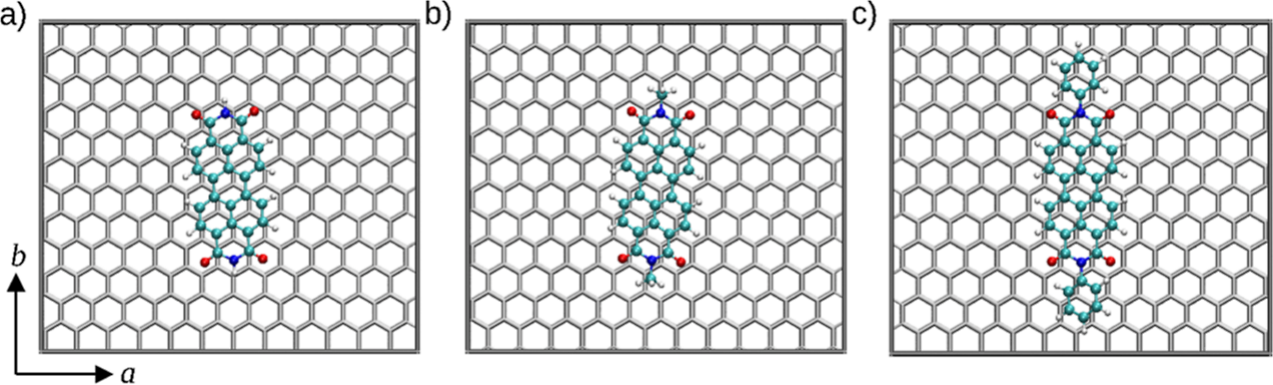
Minimum energy configurations of (a) H_2_PTCDI, (b) Me_2_PTCDI, and (c) Ph_2_PTCDI obtained via basin-hopping global optimization at the SCC DFTB/3ob level. The binding motifs show a very high similarity. The ABA stacking stands out as the preferred binding motif. The interaction energy of the systems *U*_int_ has been determined as (a) −254.3, (b) −271.9, and (c) −303.4 kJ mol^–1^, respectively. Only the first layer of the graphite carrier composed in total of four layers is shown.

To compare the electrochemical behavior of thin-film electrodes with more conventionally used counterparts, H_2_PTCDI was blended with carbon black as a conductive additive and chitosan as a binder to form a slurry. This slurry was then applied to a carbon fiber substrate, creating a film with a thickness of 15 μm. In Figure S18, the CVs of the two different electrodes are compared. The peaks described in Figure 3a are also resolved with the slurry electrode; however, two additional peak couples at 2.43/2.58 V and 1.72/1.93 V appeared. This could indicate the existence of different arrangements of H_2_PTCDI molecules in the slurry-based electrode. Furthermore, in Figure S19 GCPL measurements of the slurry-based electrode are depicted. The initial specific capacity is 111/110 mAh g^–1^ for the sodiation/desodiation, respectively. After 110 cycles, the slurry-based electrode retains 87% of its initial capacity, making it even more stable than the thin-film counterpart. Additionally, the Coulombic efficiency is nearly 100% throughout the experiment, effectively suppressing the side reactions near 1 V. Therefore, the PTCDI molecules are also applicable in a more conventional electrode design.

### Theoretical Calculations

In the majority of cases, intra- and intermolecular hydrogen bonding or/and π–π stacking interactions between neighboring planar molecular units promoted via short-ranged van der Waals contributions are regarded as the main driving force in the associated crystal formation.^70^ These interactions, particularly the π–π-stacking interaction between the pigment molecules and the carbon substrate, play a key role in the stability of the stacked layers in the PTCDI crystals, as well as in the carbon carrier substrate (i.e., graphite). It also affects the long-term stability of organic-based electrode materials, since the π–π-stacking interactions are the main driving force of the interaction between the PTCDI molecules and the graphite carrier. In this study, the structural and energetic properties of H_2_PTCDI, Me_2_PTCDI, and Ph_2_PTCDI on a graphite model system were analyzed using basin-hopping global minimization at the SCC DFTB/3ob level of theory as previously applied to the anthraquinone case.^14^ In addition, the reduced states of these substrates and the corresponding dimers were treated on a custom 2D periodic graphite system. A single substrate layer was chosen as it represents the initial step in the formation of the active thin-film material. The data obtained provides detailed insights into the interactions between substrate molecules and the surface, as well as the intermolecular interactions between the PTCDI molecules and the Na ions.

#### Binding Motif

Basin-hopping global minimization resulted in a binding motif that shows an ABA stacking order oriented parallel with respect to the *b*-axis (see Figure 8). The perylene moiety of PTCDI is characterized by extensive π–π-stacking interactions with the graphite surface, which is found to be the main driving force of the molecule–carrier interaction. H_2_PTCDI shows an average C–C distance from the graphite layer of approximately 0.31 nm. Me_2_PTCDI and Ph_2_PTCDI, on the other hand, display average distances of *z*_C–C_ = 0.32 and 0.33 nm, respectively. The binding energies of the three different substrates are given in Table 2. The binding conformation shows a clear resemblance to the ABA stacking conformation expected from a perylene system. However, the conformation deviates slightly in the actual orientation, since the conformation is oriented at a slight angle with respect to the *b* axis of the unit cell. Similarly, Me_2_PTCDI displays a conformation that is not perfectly parallel to the *b*-vector of the system. The conformation of Ph_2_PTCDI also shows a similar offset in the angle from the *b*-vector. The observed tilting of the systems can be explained by the presence of N and O atoms, as well as the methyl and phenyl residues. Consequently, the ideal ABA alignment expected for the purely aromatic lead structures perylene (C_20_H_12_) and peropyrene (C_26_H_14_) (see Figure S20 in Supporting Information) is distorted to accommodate the present functional groups in a thermodynamically favorable way. In addition, the use of a periodic environment may further promote the distortion of the ideal binding geometry. However, when considering that the overall dipole moments of the investigated compounds can be expected to be small (due to symmetry) and the fact that the distortion is also observed for the smallest system considered being H_2_PTCDI, the influence of the calculation setup can be considered as minor. It appears that an ideal alignment of the O and N atoms in the imide moieties on top of a carbon atom of the carrier is actively avoided.

**Table 2:** *U*_Int_ of the Lowest Binding Motif Exhibited for H_2_PTCDI, Me_2_PTCDI, and Ph_2_PTCDI on 2.953 × 2.558 nm Four-Layer Graphite Systems

	*U*_Int_/kJ mol^–1^
H_2_PTCDI	–254.3
Me_2_PTCDI	–271.9
Ph_2_PTCDI	–303.4

#### Reduced State

**Figure 9:**
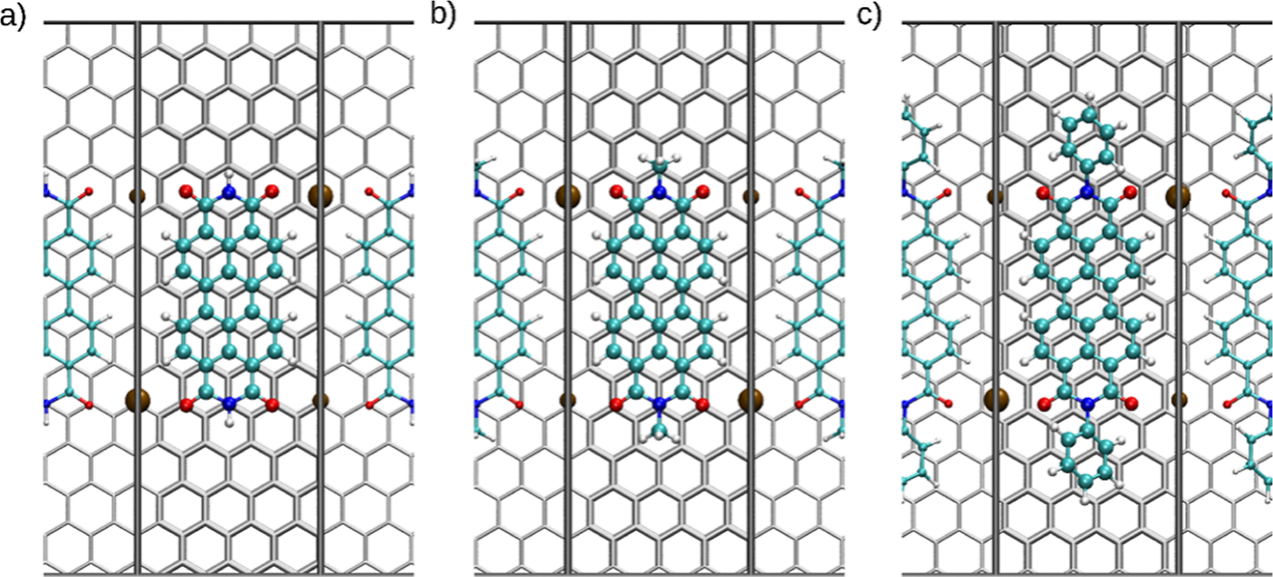
Conformation of the sodiated states of (a) H_2_PTCDI, (b) Me_2_PTCDI, and (c) Ph_2_PTCDI in a periodic cell of the size of 0.98 × 2.98 nm (4 × 14). The binding energies *U*_int_ were determined as (a) −303.7, (b) −323.3, and (c) −343.9 kJ mol^–1^, respectively.

To investigate the reduced state of the different PTCDI molecules (charge −2e^–^), different supercells were constructed with a cell length in the *x*-direction (*a*-cell vector) of 0.98 nm and therefore only variations in the *b* vector (*y* axis) of the cells are considered as demonstrated in Figure 9. Since all three molecules have the same PTCDI lead structures with an average distance between the two carbonyl oxygen atoms of *z*_O–O_ = 0.47 nm and an average Na–O distance of *z*_Na–O_ = 0.26 nm, the cell size in the a-direction was ideal to represent the monomeric state of the PTCDI molecules in the presence of Na^+^ counterions. Figure 9 shows the structures resulting from the geometry optimization. Since a periodic 2D calculation was performed, the structures shown represent a side-by-side sodiated state of the PTCDI molecules on the graphite composite.

The smallest possible cell size was found to be a 4 × 8 supercell (0.98 × 1.71 nm, see Figure S21). Using smaller cell sizes, the interaction energy increases due to an artificial interaction along the periodic *b* axis. The obtained interaction energies for H_2_PTCDI.Na_2_ and Me_2_PTCDI.Na_2_ are given in ref (3). In contrast, when the neutral molecules were added to the same 4 × 8 unit cell interaction energies of −226.5 and −189.7 kJ mol^–1^ were obtained. These findings indicate that the reduced sodiated species display a stronger interaction with the substrate carrier.

To compare all three different PTCDI derivatives, a larger cell size in the *b*-direction of 2.98 nm has been used. This was done to minimize the interaction with the neighboring PTCDI molecules along the *y* axis. Smaller graphite cell sizes have resulted in a distortion of the phenyl rings and thus nonrepresentative high energies. In Table 3, *U*_int_ is lower in the larger cell compared to the 4 × 8 systems, since the interaction between the adjacent molecules on the *b* axis is reduced with increasing distance. Compared to the other two derivatives, Ph_2_PTCDI again displays the most negative binding energy. The minimum structure of the charged Ph_2_PTCDI graphite system shows a conformation that is slightly at an angle to the *b* axis. This could result from the tilted phenyl rings that disturb the π–π stacking of the perylene subunit, since the phenyl moiety seeks a higher interaction with the graphite carrier but are hindered in rotation by the perylenetetracarboxylic substructure. The difference in the dihedral angle of the phenyl residues with respect to the PTCDI substructure in vacuum, in the crystal structure and on the graphite surface is shown in Figure S22 and listed in Table S2. It seems that the Na^+^ ions stabilize the bound conformations compared to the fully oxidized state. Interestingly, the location of the Na^+^ ions is different at the top and bottom C=O group of the substrate molecules. While on one side, the Na^+^ ion is located on the center of an aromatic ring of the carrier, it is located atop a C–C bond on the opposite side (see Figure 9). It appears that due to the alignment and the odd number of repetitions of the aromatic rings in the peropyrene lead structure the ideal locations of the Na^+^ counterions are unequally distributed on the surface. Moreover, in the case of Ph_2_PTCDI.Na_2_, again a tilt from a parallel alignment with respect to the *b* axis is observed, which further distorts the binding conformation. These results show that H_2_PTCDI and Me_2_PTCDI can be packed more densely packed on the surface than their Ph_2_PTCDI counterpart. The thin-film structure should therefore not perfectly resemble the actual crystalline structure of the diphenyl derivative when the system is charged. They more tightly resemble the actual crystalline structure of the diphenyl derivative when the system is charged. The more tightly bound state to the surface affects the binding energy by up to 35 kJ mol^–1^, but since the molecule-to-molecule distance is about the same as the crystalline distance, the bound state should be much more favorable than in the case of Ph_2_PTCDI.

**Table 3:** Sodiated State of the Reduced H_2_PTCDI, Me_2_PTCDI, and Ph_2_PTCDI on Different Four-Layer Graphite Systems −0.98 × 1.71 nm (4 × 8), 0.98 × 2.98 nm (4 × 14), 1.72 × 2.13 nm (7 × 10), and 1.72 × 2.56 nm (7 × 12)

	*U*_Int_ (4 × 8)/kJ mol^–1^	*U*_Int_ (4 × 14)/kJ mol^–1^	*U*_Int_ (7 × 10)/kJ mol^–1^	*U*_Int_ (7 × 12)/kJ mol^–1^
H_2_PTCDI.Na_2_	–283.0	–303.9	–262.6	–268.2
Me_2_PTCDI.Na_2_	–289.7	–323.3	–281.3	–290.1
Ph_2_PTCDI.Na_2_		–343.9		–310.6

**Figure 10:**
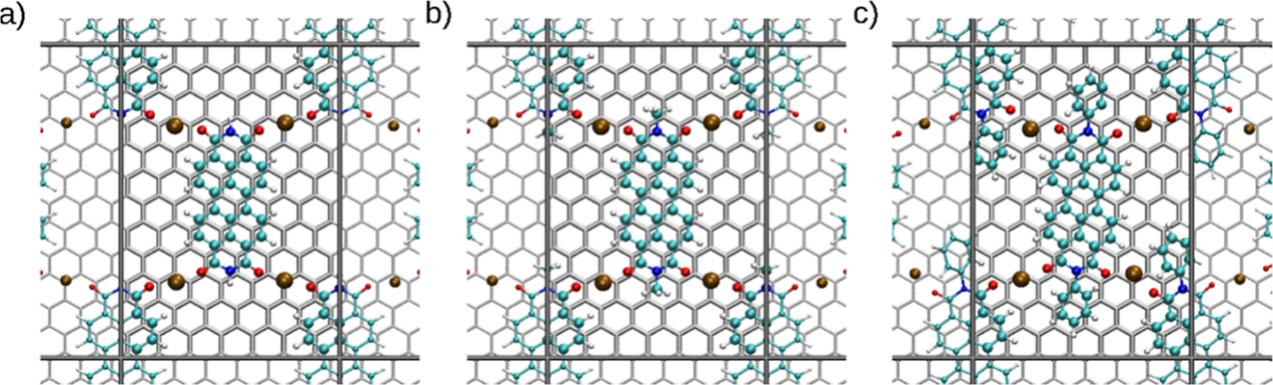
Depiction shows the diagonal conformation of (a) H_2_PTCDI, (b) Me_2_PTCDI, and (c) Ph_2_PTCDI on a 7 × 12 four-layer graphite system (1.72 × 2.56 nm). The coordination of the Na^+^ ions shows a linear coordination to the carbonyl group of the PTCDI molecules. The respective binding energies were determined as (a) −268.2, (b) −290.1, and (c) −310.6 kJ mol^–1^.

One possible conformation involving two PTCDI molecules was found to be a diagonal conformation (see Figure 10). The PTCDI molecules coordinate to the Na^+^ ions in a zigzag pattern, alternating with the upper and lower carbonyl groups of the molecule as also observed previously in the case of anthraquinone.^14^ The Na^+^ ions are coordinated at a slight angle to the carbonyl group. In Figure S23, a smaller cell size was chosen because both Me_2_PTCDI and H_2_PTCDI are much smaller in size than Ph_2_PTCDI. The conformation on the 7 × 10 graphite unit cell (1.72 × 2.13 nm) shows a wedge-shaped interaction between the Na^+^ ions and the carbonyl groups (see Figure S23). The result is a state that is almost perfectly bound to the surface, with the Na^+^ ions being located on top of an aromatic ring of the carrier. The interaction with the graphite surface of H_2_PTCDI.Na_2_ and Me_2_PTCDI.Na_2_ (3) in the diagonal conformation is smaller than the interaction calculated for the monomeric charged state. To accommodate the bulky Ph_2_PTCDI, a cell with a size of 1.72 × 2.56 nm (7 × 12) has been constructed (see Figure 10). The larger conformation stretches the coordination of the Na^+^ ions in a bonding motif that is approximately linear between the ions and the carbonyl groups. The metal ions are located on top of a carbon atom of the graphite carrier. Comparing the result to the 7 × 10 supercell (Table 3), the binding energy is lower by nearly 6–9 kJ mol^–1^. Due to the bulky phenyl rings, the binding motif does not strictly follow the ABA stacking conformation shown by the other two derivatives. This also distorts the interaction of the Na^+^ ions to a significant extent. Overall, the phenyl rings contribute significantly to the interaction energy since it shows a lower *U*_int_ by 7–16%. The dimeric conformations have a less densely packed charged state overall. Nevertheless, the binding energy is quite similar to the monomeric charged conformations. However, the diagonal charged state does not resemble the crystalline structure of the molecules and is therefore likely to affect the stability of the overall thin film.

#### Electron Mobility

**Figure 11:**
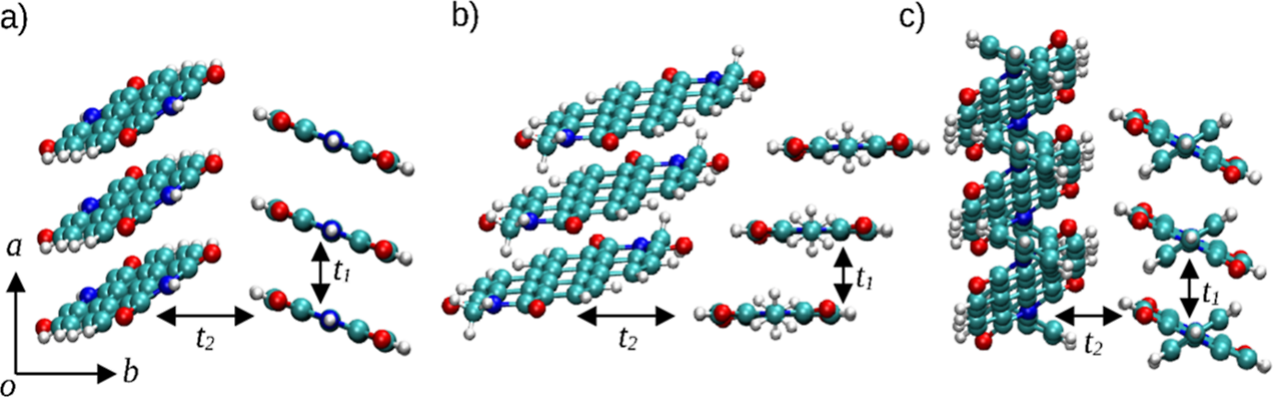
Visualization of the charge-transfer direction in (a) H_2_PTCDI, (b) Me_2_PTCDI, and (c) Ph_2_PTCDI. The directions of the π-stacking interaction and the side-by-side interaction are represented by *t*_1_ and *t*_2_, respectively.

**Figure 12:**
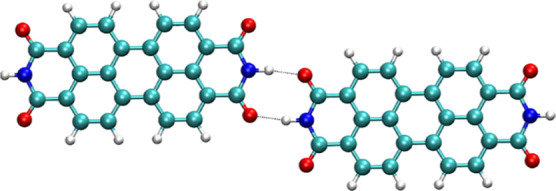
Cyclic hydrogen bond motif formed in H_2_PTCDI crystals. Binding energy Δ*E*_Bind_ is −55.6 kJ mol^–1^ or −41.8 kJ mol^–1^, calculated by DFT or DFTB, respectively.

The configurations considered in the calculation of the electron-transfer integral of H_2_PTCDI, Me_2_PTCDI, and Ph_2_PTCDI are shown in Figure 11. Both the π-stacking interaction *t*_1_ and the side-by-side interaction *t*_2_ are indicated. The resulting transfer integrals are listed in Table 4. In addition to the two directions, for the H_2_PTCDI an additional direction was considered. Two H_2_PTCDI molecules may form hydrogen bonds as shown in Figure 12, which could also lead to an additional electron-transfer direction.

**Table 4:** Transfer Integral *t* of the Interaction between Both Lowest Occupied Orbitals (LUMOs) and the Electron-Transfer Rate in Both Directions *t*_1_ and *t*_2_^a^

crystal	*t*_1_/meV	*t*_2_/meV	*k*_1_/s^–1^	*k*_2_/s^–1^	λ/kJ mol^–1^
H_2_PTCDI	29.2	17.0	2.28 × 10^12^	7.69 × 10^11^	24.9
Me_2_PTCDI	106.9	12.6	3.05 × 10^13^	4.27 × 10^11^	25.0
Ph_2_PTCDI	117.8	13.0	3.00 × 10^13^	3.68 × 10^11^	26.7

a The reorganization energy λ is also listed.

However, the transfer integral was found to be 0.8 meV, which is lower than both other direction *t*_1_ and *t*_2_ determined for the crystal configuration. Due to the presence of a N–H bond, this dimerization, leading to a cyclic motif, is only possible in the case of unsubstituted PTCDI.

In the side-by-side direction *t*_2_, H_2_PTCDI shows the highest transfer integral with 17.0 meV, compared to Ph_2_PTCDI with 13.0 meV and Me_2_PTCDI with 12.6 meV. Furthermore, the π-stacking direction *t*_1_ shows a significantly higher interaction transfer integral being nearly 1 order of magnitude larger for Me_2_PTCDI and Ph_2_PTCDI amounting to 106.9 and 117.8 meV, respectively. In contrast, H_2_PTCDI also shows a higher transfer integral *t*_2_ of 29.2 meV, but only by approximately a factor of 2 compared to the corresponding *t*_1_ value. The electron-transfer rate of both directions is not only influenced by higher transfer integral but also by lower interaction energy. The overall fastest electron transfer has been found to be in the π-stacking interaction of Me_2_PTCDI of 3.05 × 10^13^ s^–1^ with Ph_2_PTCDI showing an almost equal value of 3.00 × 10^13^ s^–1^. In contrast, H_2_PTCDI shows a significantly slower electron-transfer rate than the above-mentioned derivatives. Additionally, the electron–hole transport, which is calculated by the transfer integral between the HOMOs, has also been calculated and is listed in Table S3.

## Discussion

Based on the result of the theoretical calculations, employing the basin-hopping global optimization approach, an ABA stacking conformation is the preferred interaction motif of the PTCDI molecules bound to the graphite surface. The molecules in the bound state on the carrier resemble the underlying stacking conformation of graphite. Therefore, the π–π stacking interaction is the main driving force of the interaction between the pigments and carrier as expected. The interaction parameters *G* close to 2 for the neutral PTCDI films in close proximity to the carbon fiber substrate (*G* for peak A_H_, H_2_PTCDI, A1_Me_, Me_2_PTCDI, and A2_Ph_, Ph_2_PTCDI, Figure 4) demonstrate the strong attractive interactions between the carbon fiber substrate and the PTCDI molecules. However, the electrochemical experiments may suggest that the bulk of Me_2_- and Ph_2_PTCDI molecules are adsorbed in a configuration that differs from the layer in close proximity to the carbon fiber substrate. This is illustrated by the additional CV peaks A2_Me_ for Me_2_PTCDI (Figure 3b) and A1_Ph_ and A3_Ph_ for Ph_2_PTCDI (Figure 5) appearing at slow scan rates. However, the H_2_PTCDI molecules seem to be adsorbed in a single configuration. From the theoretical calculations, the Ph_2_PTCDI and Me_2_PTCDI molecules are found to be adsorbed more strongly (and they may be bound stronger in the adjacent layers to the carbon fiber substrate). However, the interaction of the Me_2_- and Ph_2_PTCDI molecules with the carbon fiber substrate seems to generate a layer conformation which is unfavorable for the formation of thicker films. Therefore, molecules of a certain distance away from the carbon fiber substrate form a different configuration, which is adsorbed much weaker highlighted by the rapid loss of active material of Me_2_- and Ph_2_PTCDI during cycling (see Figure 7). Furthermore, the electron transfer between this configuration and the carbon fiber substrate and/or the adjacent layer seems to be kinetically hindered. Additionally, the Ph_2_PTCDI layer adjacent to the carbon fiber substrate seem to undergo structural rearrangements during reduction and back-oxidation (Figure 6), indicating a different binding motif in the presence of Na ions. This is observed as a tilt from a parallel alignment with respect to the *b* axis in the charged state in the theoretical calculations (see Figure 9). The main driving force for the stability of the thin film seems to be a combination of the interaction of the first thin-film layer with the graphite carrier and the similarity with the crystal structure of the PTCDI molecules. Overall, the H_2_PTCDI molecule exhibits the most favorable binding motif. It shows a strong interaction while also displaying minimal deviation from the crystal structure when bound to the graphite surface. In addition, the difference between the neutral and charged states on the 4 × 8 unit cell is much smaller. This indicates higher stability throughout the cycle of the cathode material, since the difference in interaction energy could promote the generation of excess heat, potentially promoting a decomposition of the thin-film structure. Experimentally, this is highlighted by the more stable gravimetric capacity of the H_2_PTCDI film during cycling (Figure 7).

The electron-transfer rates determined based on the crystal configurations (see also Figure S24 for calculated XRD patterns) were found to be significantly larger for the substituted PTCDI derivatives, suggesting that phenyl and methyl groups contribute meaningfully to a higher interaction of the LUMOs. However, the experimental data obtained for the thin-film batteries suggest that unsubstituted H_2_PTCDI displays higher electron mobility than the substituted counterparts. One possible explanation could be that the electron mobility of the cathode material is not limited by the mobility in the crystal solid but by the mobility between the carbon fiber substrate and the first layer of PTCDI molecules, and in the case of Me_2_- and Ph_2_PTCDI, by the electron transfer between the adjacent layer and the bulk. Moreover, unsubstituted PTCDI can form H-bonds in between molecules, resulting in a cyclic H-bonded interaction motif. While it can be expected that such an interaction is influencing the electron-transfer properties, the associated system size, comprised of four PTCDI molecules, results in highly demanding DFT calculations, that were at present not feasible. However, it can be expected that this particular interaction between the PTCDI molecules can also enhance the interaction with the carbon carrier, which could be an explanation for why H_2_PTCDI exhibits higher stability during charge–discharge cycles of the cathode material since the crystal structure on the graphite support is in a more energetically favorable conformation.

## Conclusion

Three different PTCDI derivatives were investigated as PTCDI carbon fiber composite electrodes in sodium-ion battery half-cells. Theoretical calculations concerning the adsorption of the molecules on top of the carbon fiber substrate in their neutral and reduced state and their electron mobility in the crystal structure were done. It is revealed that the thermal evaporated PTCDI films on top of the carbon fiber substrate behave as surface-confined systems with a diffusion-less mechanism in which the diffusion of the sodium counterions is not rate-limiting. The PTCDI films exhibit strong attractive interaction forces between the PTCDI molecules and between the PTCDI molecules and the carbon fiber substrate. H_2_PTCDI molecules mostly form a homogeneous film on top of the carbon fiber substrate, exhibiting the typical electrochemical behavior of surface-confined systems in which attractive interactions are prevalent. Me_2_PTCDI molecules adjacent to the carbon fiber substrate show a different electrochemical behavior compared to the bulk of Me_2_PTCDI. It is revealed that the redox reaction of the bulk is mediated through an electron exchange reaction between the Me_2_PTCDI layer adjacent to the carbon fiber substrate and the bulk. This electron exchange reaction is found to be slow, giving rise to a diffusion-like controlled process. Ph_2_PTCDI molecules are found to be adsorbed at two different arrangements on top of the carbon fiber substrate. Arrangement 1 exhibits a concerted two electron redox reaction in which the structure changes upon reduction/back-oxidation to a configuration which is appropriate for its charged state. The redox reaction of arrangement 2 is kinetically hindered. Furthermore, for small coverages, arrangement 1 is the preferred structure of adsorbed molecules, whereas with an increasing film thickness, the molecules assemble in arrangement 2. It is revealed that the substitution of the hydrogen residual with bulky side groups, like methyl and phenyl, shifts the redox potential of the carbon fiber composite electrode in the anodic direction, making the film easily reducible. Additionally, the loss of active material during electrochemical cycling is elevated by exchanging the residual from hydrogen to bulky side groups. These findings highlight the influence of structural properties on the electrochemical behavior of organic molecules. It also exposes the need for structural integrity during electrochemical cycling to avoid the loss of active material. While pristine PTCDI carbon fiber composite electrodes show very promising electrochemical properties, like gravimetric capacities near their theoretical value in GCPL experiments and very fast counterion diffusion into, out of and through the film, the loss of active material due to the inhomogeneity of the adsorbed films is a concern. Strategies to avoid the loss must be developed, possibly through further modifications of the PTCDI framework or by finding substrates which enhance the stability and integrity of the adsorbed films.

## Supporting Information Available

Methodology of binding energy calculation; structure and the proposed redox reaction of the PTCDI molecules; Raman spectrum of the carbon fiber substrate; experimental and calculated Raman wavenumbers; CV of the carbon fiber substrate; CVs of H_2_-, Me_2_-, and Ph_2_PTCDI with different scan rates; log(peak current) versus log(scan rate) plots; charge stored for Me_2_PTCDI; CV of Me_2_PTCDI with different film thicknesses; peak potential versus ln (scan rate) plots; optical images of Ph_2_PTCDI films; CV of H_2_PTCDI with different film thicknesses; differential capacity plot of H_2_PTCDI; comparison of CVs of thin film and slurry-based electrodes; specific capacities and Coulombic efficiencies of a slurry-based H_2_PTCDI electrode; lead structures; conformation of the sodiated state of H_2_PTCDI^2–^ and Me_2_PTCDI^2–^ in an 4 × 8 supercell; side view of Ph_2_PTCDI showing the conformational distortion of the phenyl rings; dihedral angle; diagonal conformation of H_2_PTCDI and Me_2_PTCDI in the sodiated state on a 1.72 × 2.13 nm (7 × 10) graphite layer; calculated XRD patterns of H_2_PTCDI, Me_2_PTCDI, and Ph_2_PTCDI using Cu Kα radiation at 1.540598 angström; transfer integral *t* of the HOMO–HOMO interaction and the electron–hole transfer rate *k* for both directions *t*_1_ and *t*_2_; and reorganization energy λ (PDF)
