# Supplementary material for: Perylenetetracarboxylic Diimide Composite Electrodes as Organic Cathode Materials for Rechargeable Sodium-Ion Batteries: A Joint Experimental and Theoretical Study
Source: ACS Omega. 2024 Jan 31;9(6):6642–57. doi: 10.1021/acsomega.3c07621 (PMC10870290; doi:10.1021/acsomega.3c07621)
Supplement: Supplementary file 1 — ao3c07621_si_001.pdf [file ao3c07621_si_001.pdf]

Supporting Information

Perylenetetracarboxylic Diimide (PTCDI) Composite Electrodes as Organic Cathode Materials for Rechargeable Sodium-Ion Batteries: A Joint Experimental and Theoretical Study

Sebastian Liebl, [a]‡ Josef M. Gallmetzer, [c]‡ Daniel Werner, [a] Dogukan H. Apaydin, [b] Thomas S. Hofer, [c]* and Engelbert Portenkirchner[a]*

[a] *S. Liebl, D. Werner, E. Portenkirchner*

Institute of Physical Chemistry

University of Innsbruck

6020 Innsbruck (Austria)

[b] *D. H. Apaydin*

Institute of Materials Chemistry

Vienna University of Technology

1060 Vienna (Austria)

[c] *J. M. Gallmetzer, T. S. Hofer*

Institute of General, Inorganic and Theoretical Chemistry

University of Innsbruck

6020 Innsbruck (Austria)

Methodology Binding Energy Calculation

A four-layer graphite supercell was constructed to study the ideal binding motif, taking the 2D periodicity of the system into account. The cell parameter c along the z-axis was set to 10 nm. First, a basin-hopping approach[1] was used to compute the ideal binding motif on the graphite surface, as performed in a previous work on anthraquinone (AQ) bound to a graphite surface.[2] This approach provides a discretization of the underlying potential energy surface (PES), which enables the identification of the ideal binding motif of the adsorbate on the surface. The scan was performed with a single layer, that had a unit cell length along the orthogonal a and b axis of 2.953 x 2.558 nm, which would correspond to a 12x12 supercell. The scan consisted of 15 angular increments in the range of [-30°, 120°] and five increments along the a-axis in the range of [0 nm, 0.123 nm]. The range of the increments along the a-axis is half of the cell size a_{graph} of 0.2504 nm, which corresponds to the length of one C2 unit of the minimized graphite structure. After the scan, the conformation with the lowest binding energy was further refined by adding three additional layers to the system, which results in an improved estimation of the binding energy of the adsorbate on the graphite surface. The use of four layers in this work represents a compromise between estimating the binding energy and limiting the computational cost of the system. Previous work on the AQ-graphite interaction,[2] considering one to six layers, has shown that a four-layer system already gives reasonable results for the binding energy. In this study, only a four-layer system was considered to keep the computational costs manageable.

Subsequently, Na-ions were added, to the obtained binding motif, to investigate the sodiated state of the cathode material. These calculations were carried out considering a monomeric and a range of possible dimeric binding motifs. For the dimer structures, a

second unit of the active molecules was arranged similar to the ideal binding motif determined in the previous step. To find the ideal binding motif and to compare the interaction energies of the three distinct adsorbates, a range of different system size for the graphite lattice had to be considered.

The binding energy was calculated for both the sodiated and desodiated states. The associated binding energy U_{int} is calculated via

$$U_{\mathrm{int}} = U_{\mathrm{system}} - (U_{\mathrm{surf}} + n_{\mathrm{mol}} * U_{\mathrm{mol}})$$

, where U_{mol} is the energy of the nmol molecular species, Usurf is the energy of the four-layer graphite system and U_{system} is the total energy of the PTCDI-graphite system, all determined using the same periodic calculation setup. U_{int} not only account for the interaction energy between the surface to the adsorbate, but in the case of the dimer structures, also for the interaction between the considered adsorbates.

Redox Reaction:

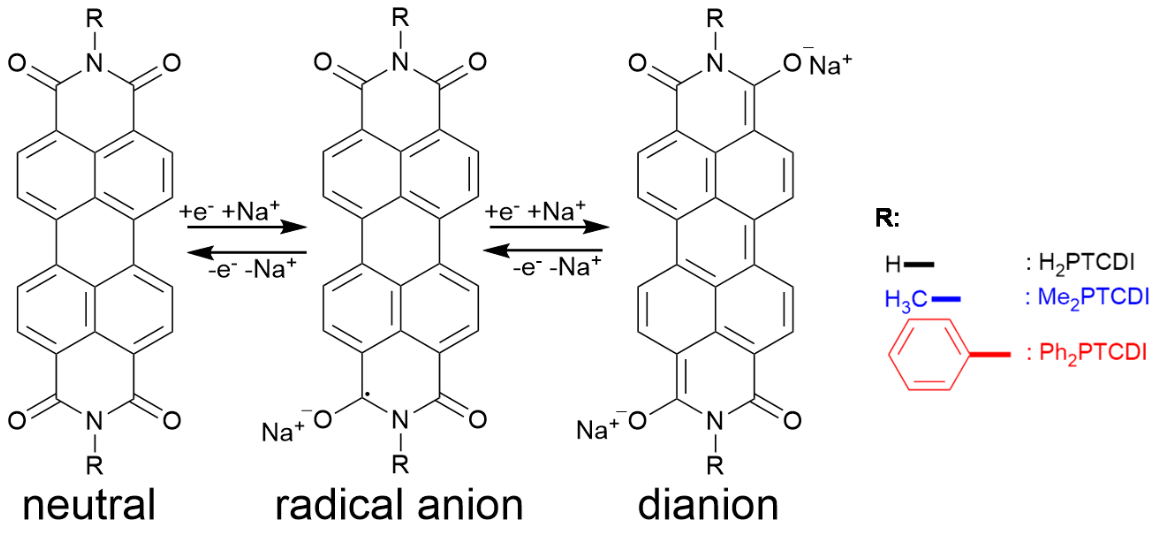

Figure S1. Molecular structure and proposed redox reaction of the PTCDI molecules.

Raman Spectrum Carbon Fiber Substrate

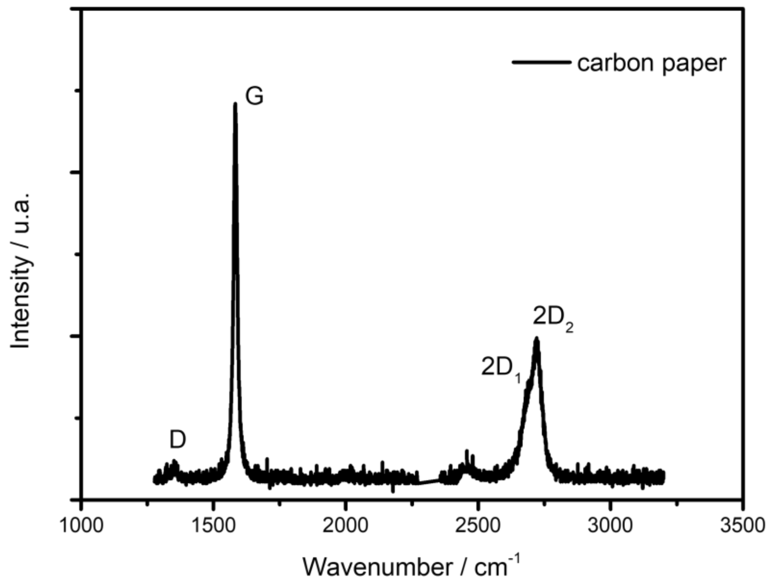

Figure S2. Raman spectrum of pure carbon paper. The peaks are denoted in their typical notation.

Raman Wavenumbers:

Table S1. Experimental and calculated Raman wavenumbers in cm^{-1}:

H_2		Me_2		Ph_2		
Exp.	Calc.	Exp.	Calc.	Exp.	Calc.	**Interpretation**
247	250	224	234	161	233	C-N-C scissor
		444				δ(C-C), oop
550	548	541	539	546	546	lateral stretch whole molecule
		571	573			δ(CH_3)
652	655			637		central ring stretch
		1294	1307	1298	1308	δ(CH)
1306	1322	1306	1326	1308	1328	central ring stretch + δ(CH)
1381	1397	1385	1416	1385	1394	δ(C–H) + ν(C–C) + δ(CH_3)
1451	1473	1461	1484	1457	1478	ν(C–C)
1574	1606	1574	1607	1576	1607	δ(C–H) + ν(C–C)
1588	1629	1591	1625	1590	1625	δ(C–H) + ν(C–C)
1617	1654	1617	1654			ν(C–C)

CV of the Carbonfiber Substrate:

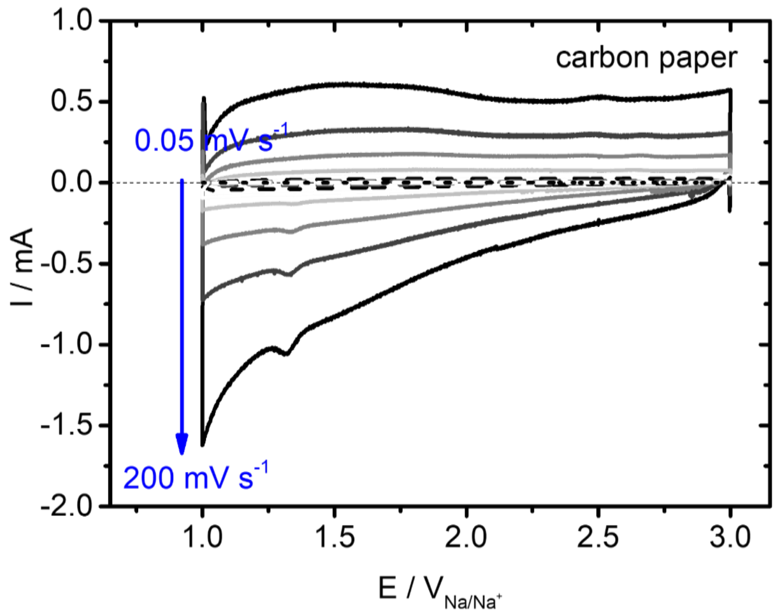

Figure S3. CV of the pure carbon fiber substrate measured at the scan rates from 200 to 0.05 mV s^{-1}.

CV of PTCDI derivatives with different applied scan rates:

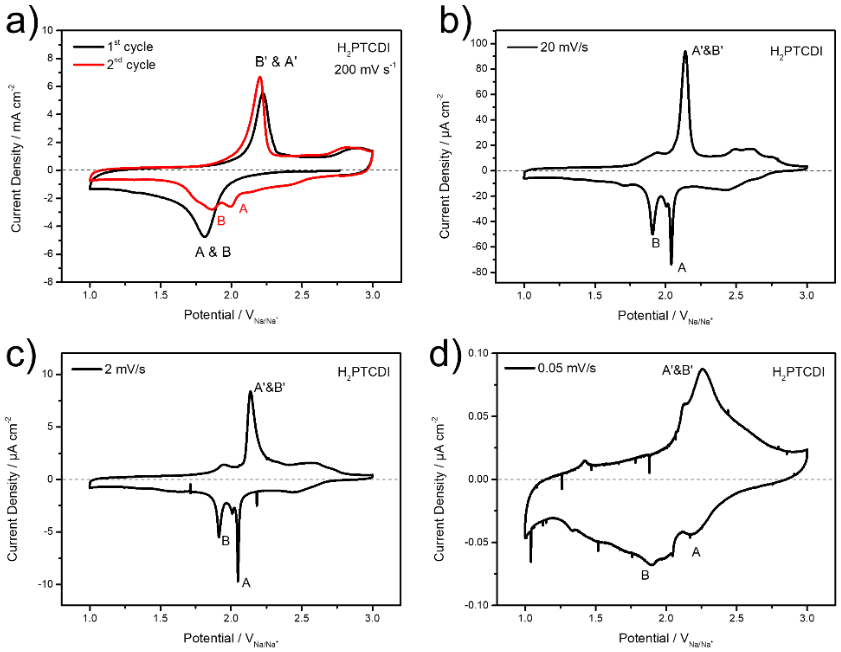

Figure S4. CV of a 250 nm thick H_2PTCDI film: a) first two cycles with a scan rate of 200 mV s^{-1} b) 20 mV s^{-1} c) 2 mV s^{-1} d) 0.05 mV s^{-1}.

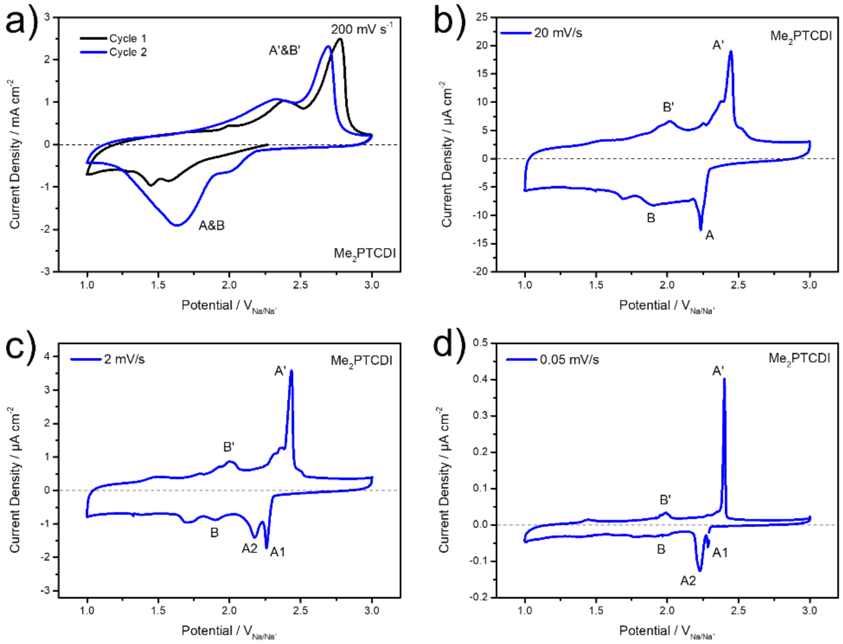

Figure S5. CV of a 250 nm thick Me_2PTCDI film: a) first two cycles with a scan rate of 200 mV s^{-1} b) 20 mV s^{-1} c) 2 mV s^{-1} d) 0.05 mV s^{-1}.

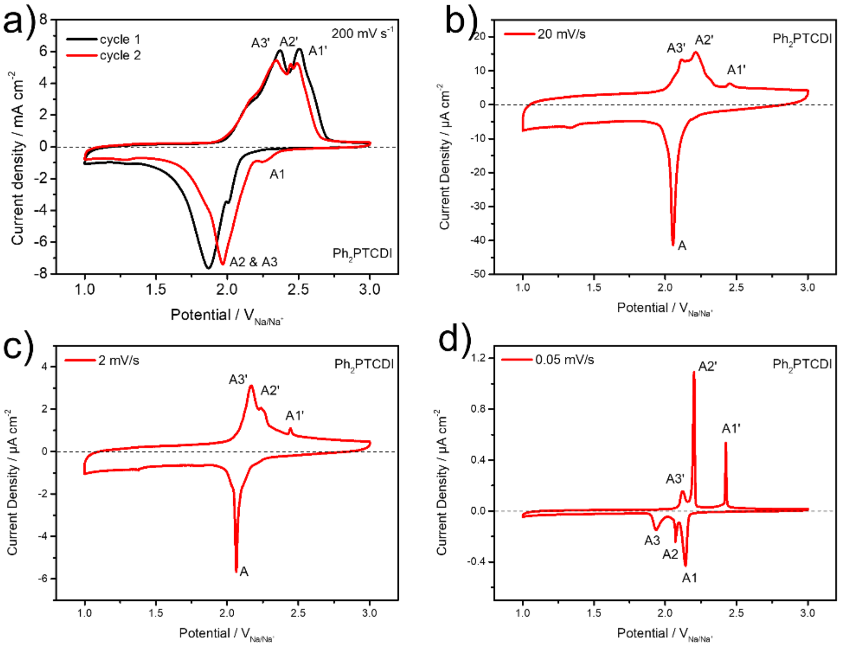

Figure S6. CV of a 250 nm thick Ph_2PTCDI film: a) first two cycles with a scan rate of 200 mV s^{-1} b) 20 mV s^{-1} c) 2 mV s^{-1} d) 0.05 mV s^{-1}.

Log (Peak Current) versus Log (Scan Rate) plots:

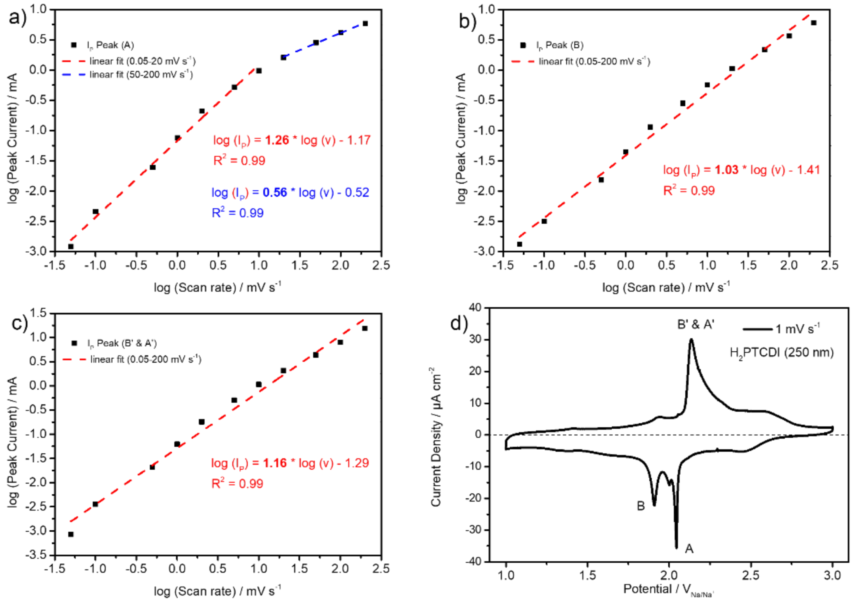

Figure S7. Log (peak current) versus log (scan rate) plots with linear fit. a) Peak A b) peak B c) peak B' & A' d) CV of H_2PTCDI with a scan rate of 1 mV s^{-1}.

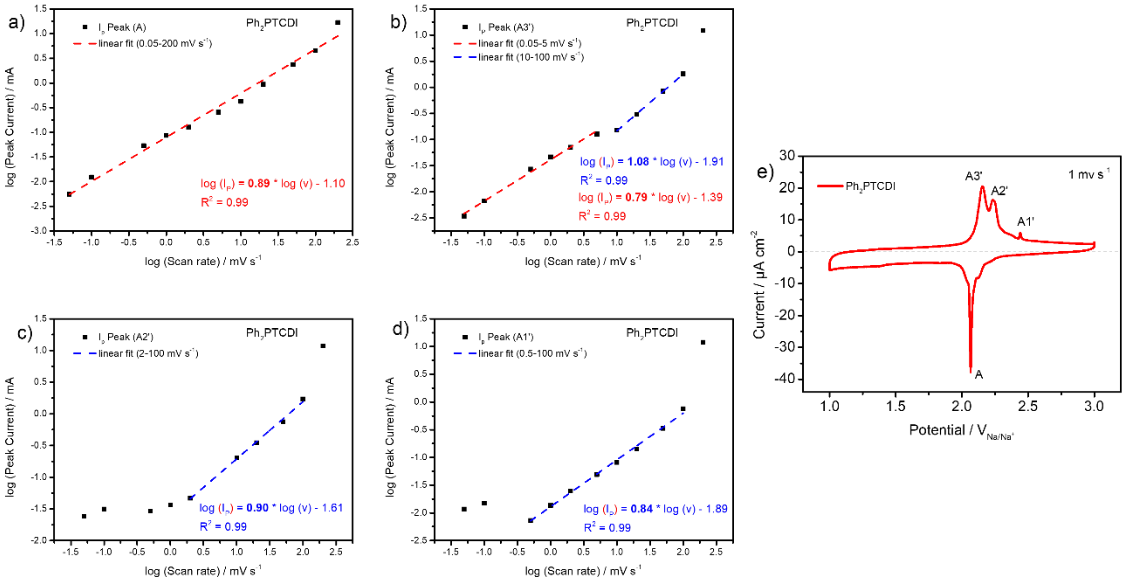

Figure S8. Log (peak current) versus log (scan rate) plots with linear fit. a) Peak A b) peak A3‘ c) peak A2‘ d) peak A1‘ e) CV of H2PTCDI with a scan rate of 1 mV s^{-1}.

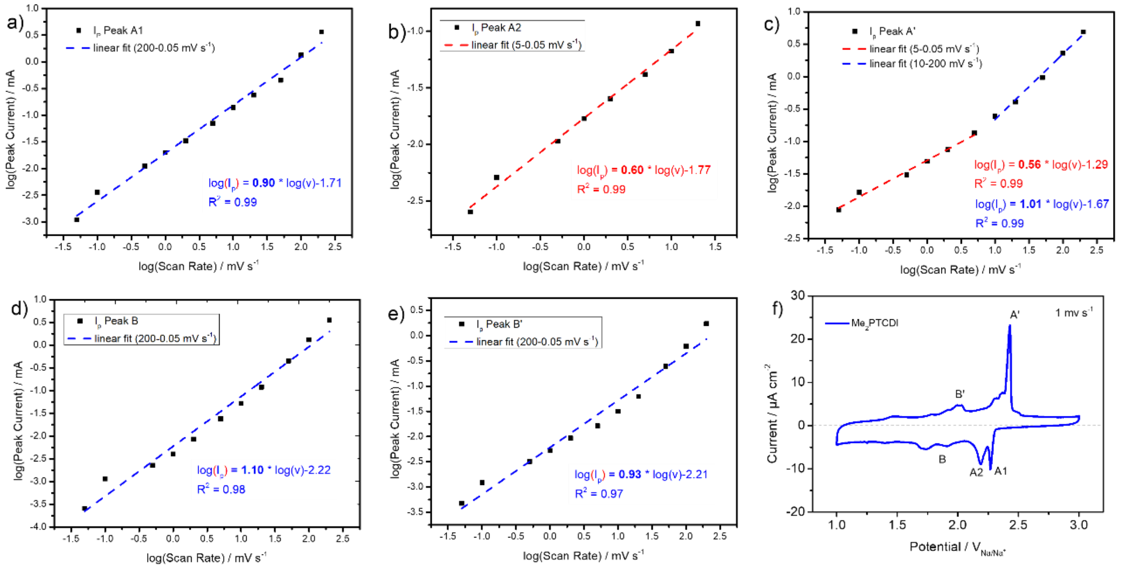

Figure S9. Log (peak current) versus log (scan rate) plots with linear fit. a) Peak A1 b) peak A2 c) peak A‘ d) peak B e) peak B‘ f) CV of Me2PTCDI with a scan rate of 1 mV s^{-1}.

The values of the peak currents I_P are the absolute values of the respective CV measurements, with the capacitive current measured for the pure carbon paper substrate without active material subtracted.

Charge stored Me_2PTCDI:

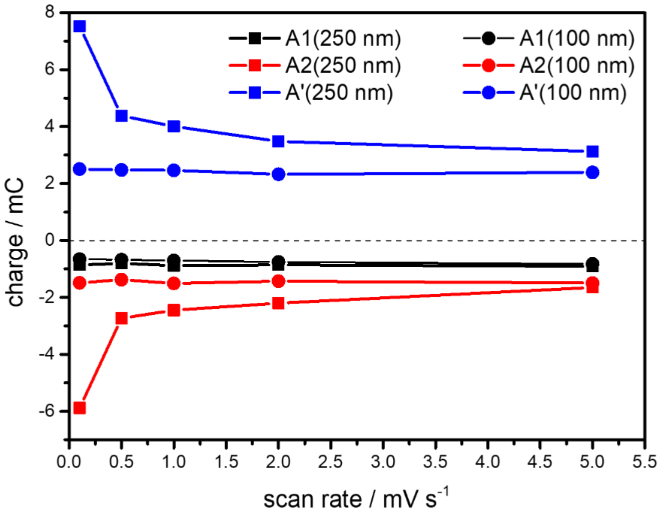

Figure S10. Dependence of charge conversion of the peaks A1 (black), A2 (red) and A' (blue) on the scan rate for a 250 nm (squares) and a 100 nm (circles) Me_2PTCDI composite electrode. Lines are a guide for the eye and have no physical meaning.

CV of Me_2PTCDI with different film thicknesses:

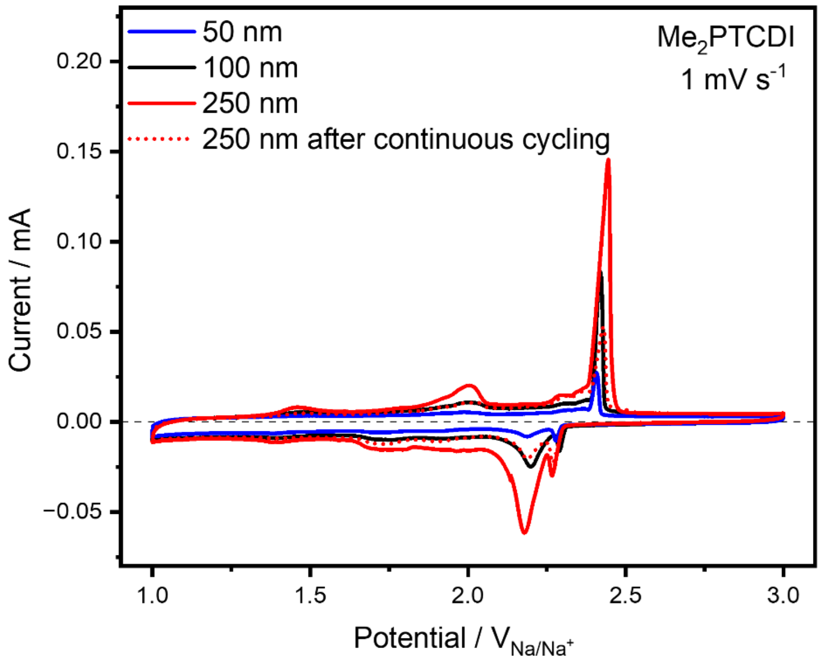

Figure S11. CVs of Me_2PTCDI with a film thickness of 50 nm (blue), 100 nm (black) and 250 nm (red) and a scan rate of 1 mV s^{-1}.

Peak Potential versus ln (scan rate) plots:

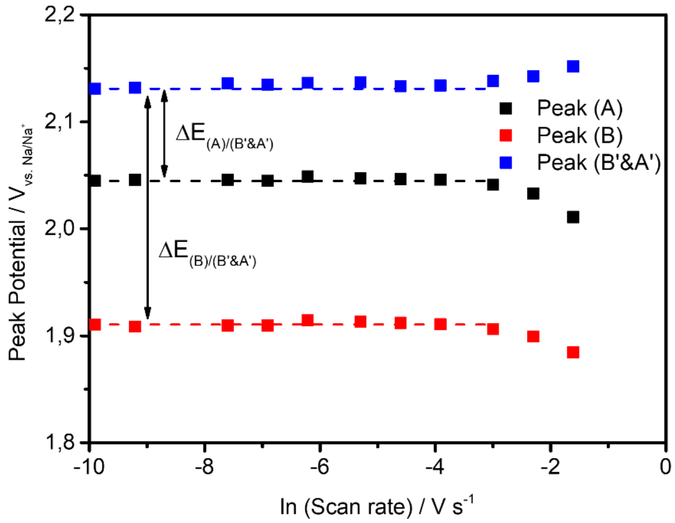

Figure S12. Peak potentials plotted against the (ln) of applied scan rates. Black squares are the values for the reduction peak A. Red squares are the values for the reduction peak B. Blue squares are the values for the back-oxidation peak B'&A'. Dashed lines are guidance for the eyes only. The difference between peak potentials are indicated as $\Delta E_{(A)/(B'\&A')}$ *and* $\Delta E_{(B)/(B'\&A')}$ *for the difference between the reduction peak A and the back-oxidation peak B'&A' and for the difference between the reduction peak B and back-oxidation peak B'&A', respectively.*

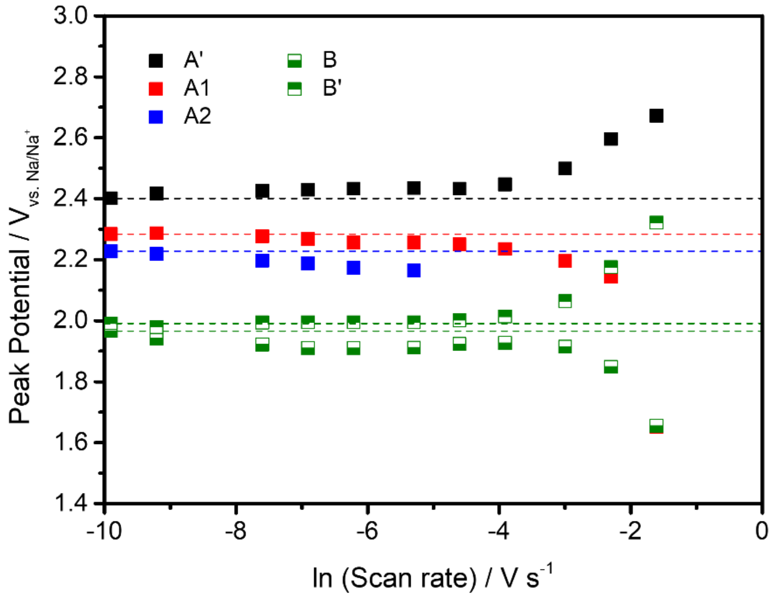

Figure S13. Dependence of the peak potential for the reduction peaks A1 (red squares), A2 (blue squares) and B (bottom-filled green squares) and the back-oxidation peaks B' (top-filled green squares) and A' (black squares) on the scan rate of Me_2PTCDI*. Dashed lines indicate the peak potentials at the slowest scan rate (0.05 mV* s^{-1}*).*

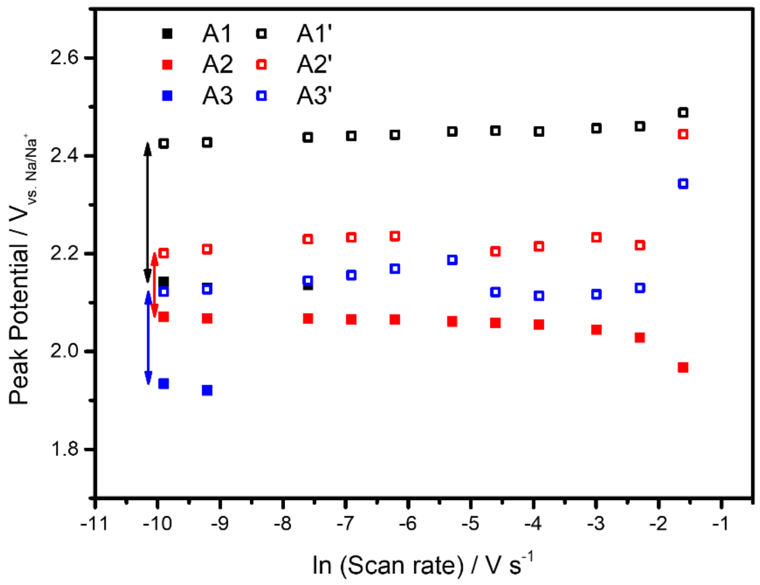

Figure S14. Peak potentials plotted against the (ln) of applied scan rates. The reduction peaks A1(black), A2(red) and A3(blue) are depicted as filled squares. The back-oxidation peaks A1'(black), A2'(red) and A3'(blue) are depicted as empty square. The double-sided arrows indicate the peak potential differences between A1 and A1' (black), A2 and A2' (red), A3 and A3' (blue), respectively.

Optical images of Ph_2PTCDI films:

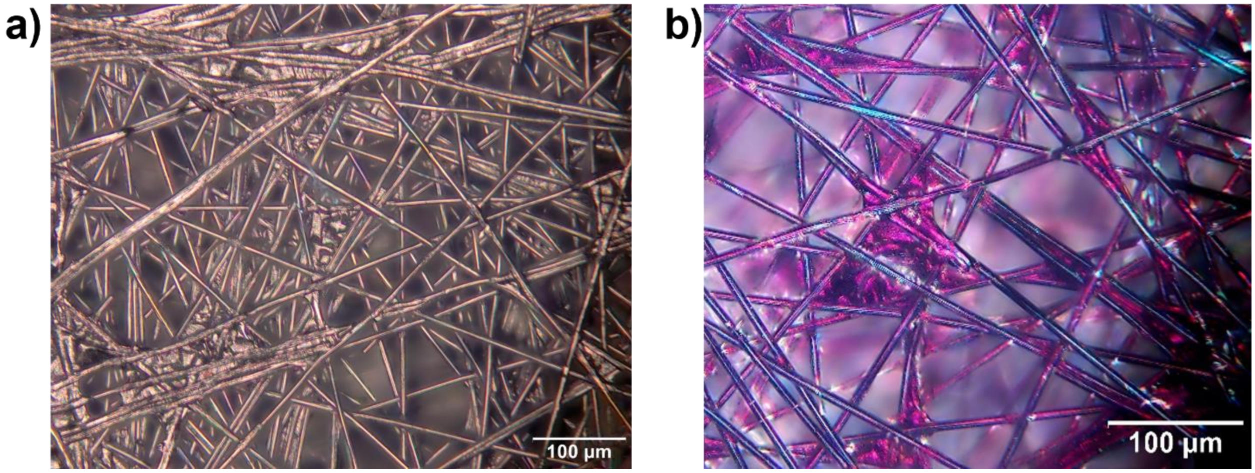

Figure S15. a) Optical image of a 100 nm thick pristine Ph_2PTCDI electrode. b) Optical image of a 50 nm thick pristine Ph_2PTCDI electrode. The magnification is 20x.

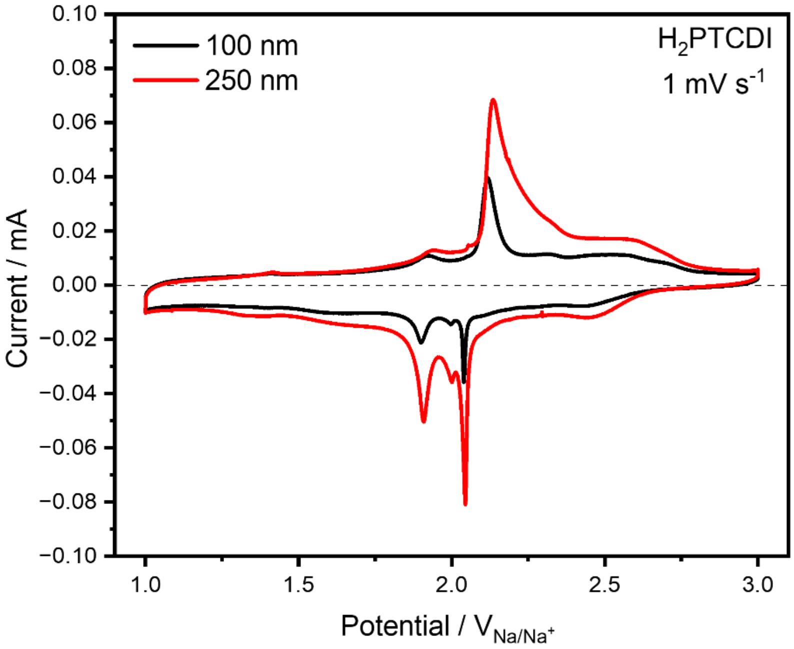

Figure S16. CVs of H_2PTCDI with a film thickness of 100 nm (black) and 250 nm (red) and a scan rate of 1 mV s^{-1}.

Differential Capacity Plot of H_2PTCDI:

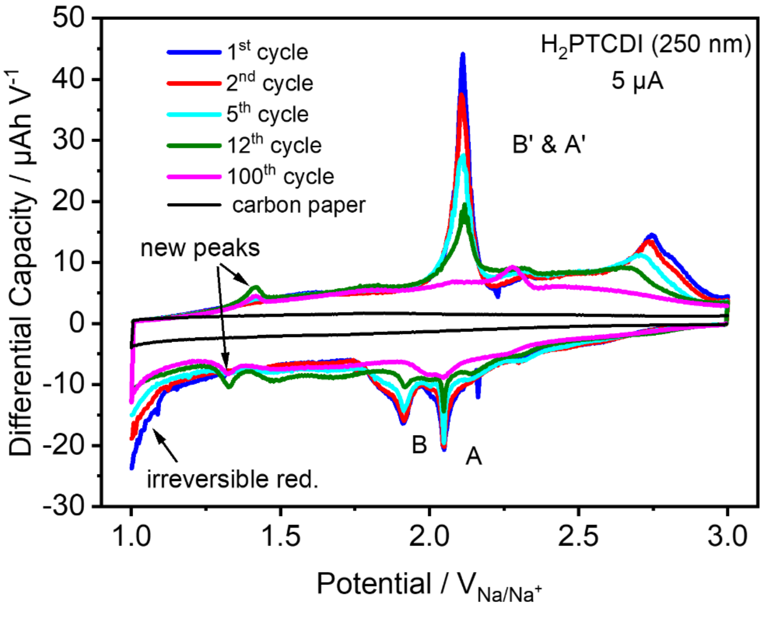

Figure S17. Differential capacity plots for the GCPL data obtained with an applied constant current of 5 µA. Course of the differential capacity of an initial 250 nm H_2PTCDI composite electrode from its first to its 100th cycle. New additional peaks are highlighted with arrows. The differential capacity plot of carbon paper is illustrated as dashed line.

Comparison of CVs of thin film and slurry-based electrodes:

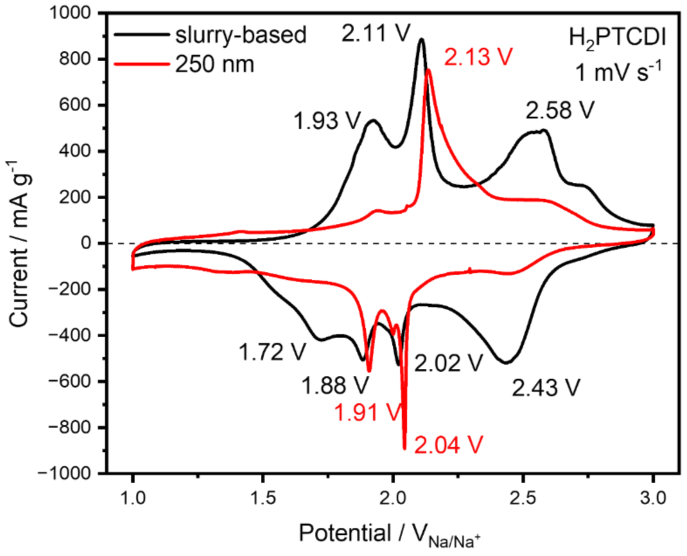

Figure S18. CVs of a 250 nm thick H_2PTCDI film (red) and a slurry-based H_2PTCDI electrode (black) with a composition of 55% active material 35% carbon black and 10% chitosan. The mass of active material for the slurry-based electrode is 1.65 mg. The scan rate used is 1 mV s^{-1}. Peak potentials are given with the respective color.

Specific capacities and coulombic efficiencies of a slurry-based H_2PTCDI electrode:

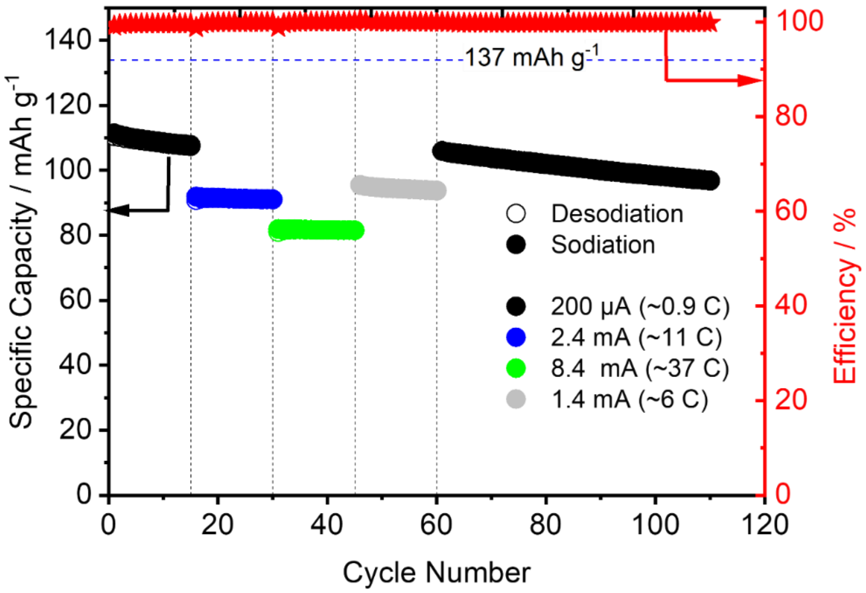

Figure S19. Specific capacities of a slurry-based H_2PTCDI electrode with a composition of 55% active material 35% carbon black and 10% chitosan. The mass of active material for the slurry-based electrode is 1.65 mg. The different applied constant currents are 200 µA (black), 2.4 mA (blue), 8.4 mA (green) and 1.4 mA (grey). The coulombic efficiency is depicted in red.

Lead Structures:

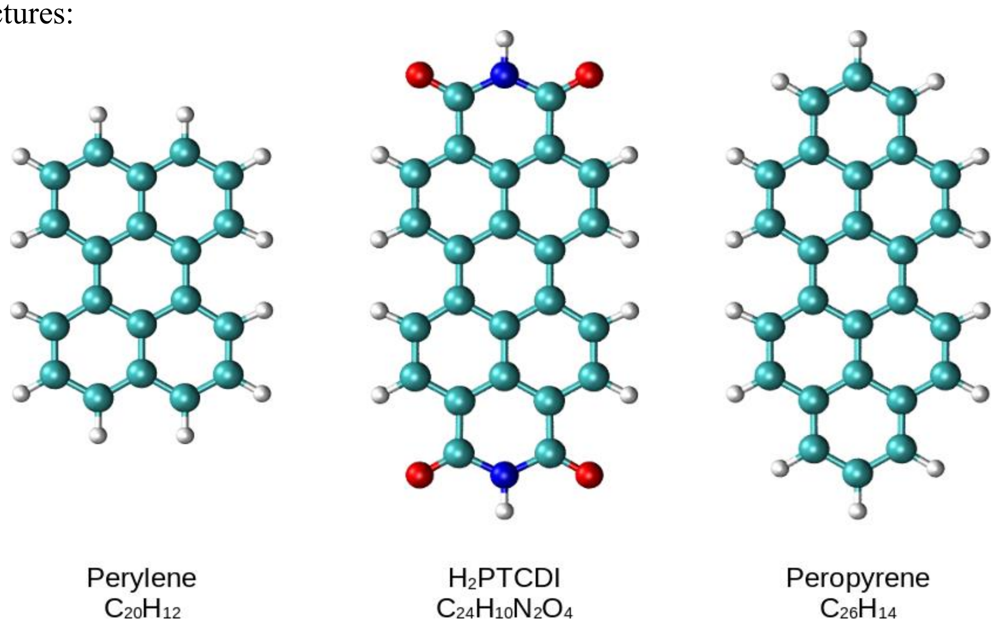

Figure S20. Depiction of the molecular structure of H_2PTCDI ($C_{24}H_{10}N_2O_4$, center) and the associated lead structures being perylene ($C_{20}H_{12}$, left) and peropyrene ($C_{26}H_{14}$, right).

Conformation of the PTCDI molecules:

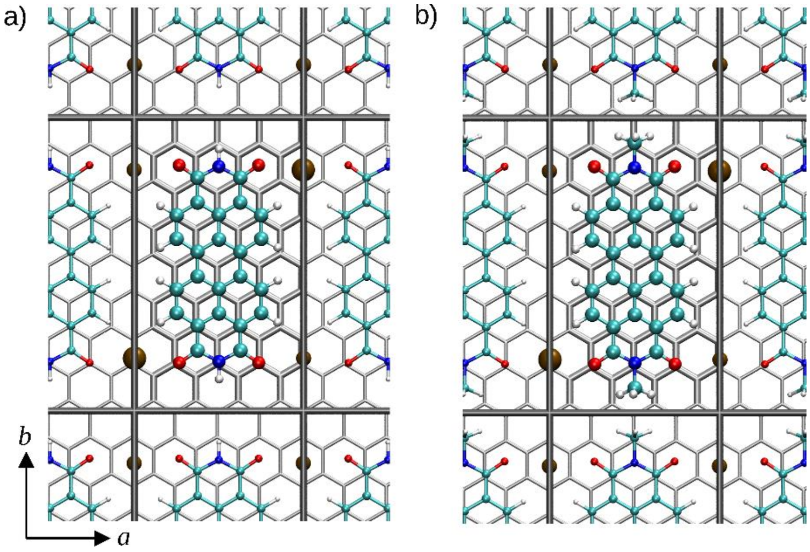

Figure S21: Conformation of the sodiated state of (a) H_2PTCDI^{2-} and (b) Me_2PTCDI^{2-} in an 4x8 supercell, which corresponds to a size of 0.98x1.71 nm. The respective binding energies were determined as (a) -283.0 and (b) -289.7 kJ mol^{-1}.

Dihedral angle:

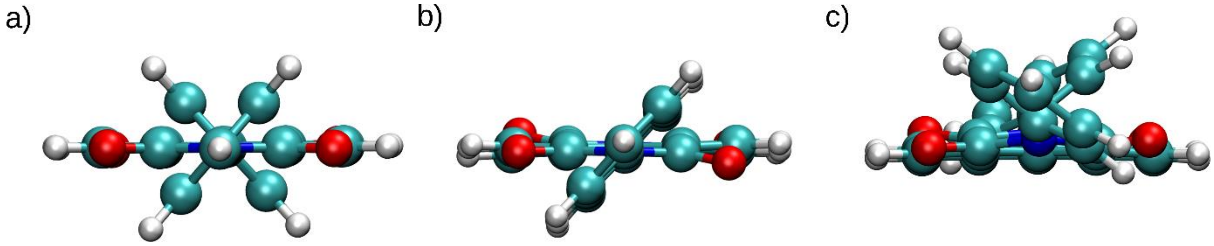

Figure S22: Side view of Ph_2PTCDI showing the conformational distortion of the phenyl rings with respect to the PTCDI substructure. The different conformations result from an SCC DFTB/3ob level of theory calculation a) in vacuum, b) in the crystal (bulk) and c) on a four-layer graphite surface.

Table S2: Dihedral angle (CN(PTCDI)-CC(Ph)) of the phenyl ring with respect to the PTCDI substructure in vacuum, bulk and on a four-layer graphite surface. The calculations have been performed with SCC DFTB/3ob level of theory.

	Dihedral / °
Vacuum	49.1
Bulk	47.4
Surface	35.9

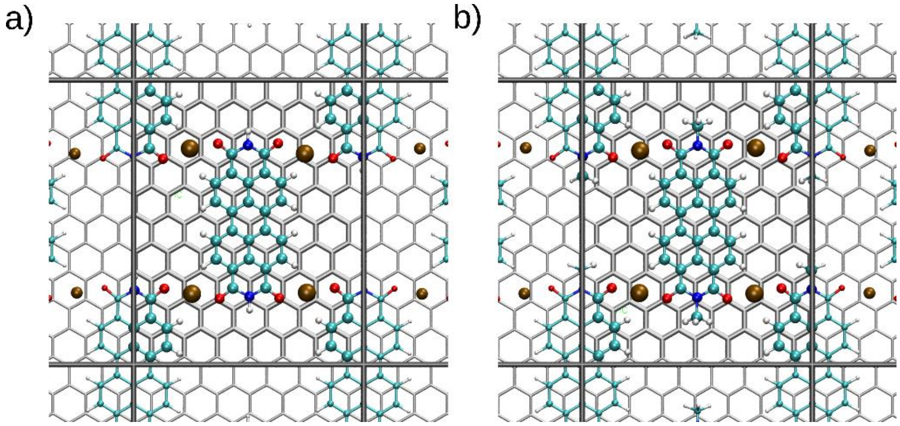

Figure S23: Diagonal conformation of a) H_2PTCDI and b) Me_2PTCDI in the sodiated state on a 1.72x2.13 nm (7x10) graphite layer is shown. The coordination of the Na^+-ion shows a wedge-shape to the carbonyl group of the PTCDI molecule. The interactions energy U_{int} has been determined as a) -262.6 and b) -281.3 kJ mol^{-1}, respectively.

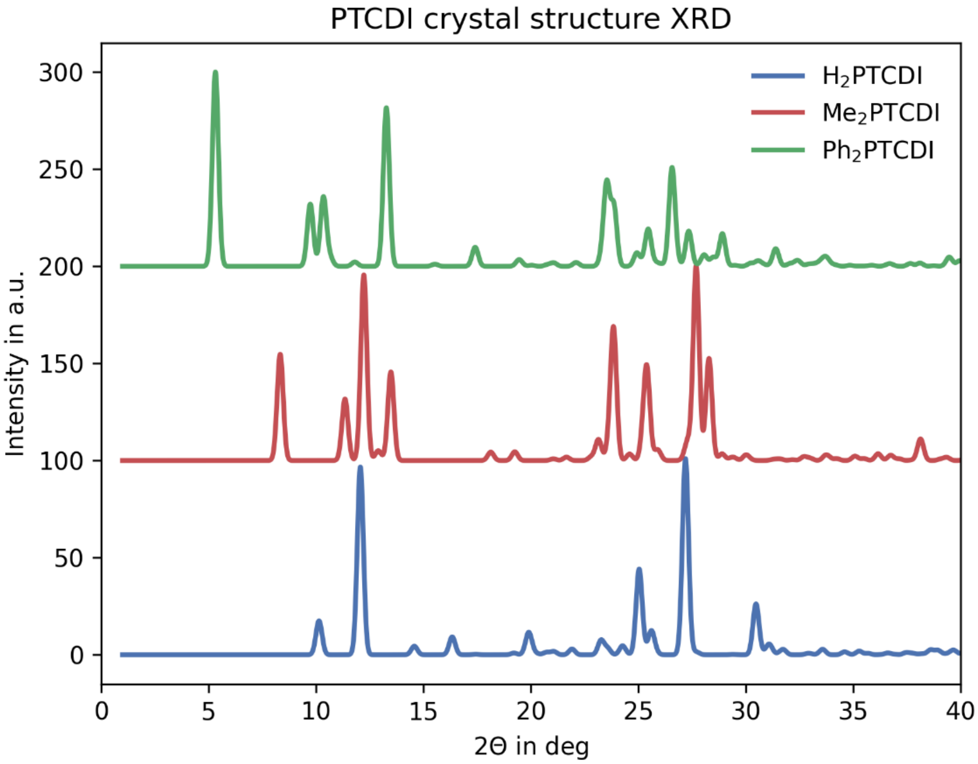

Figure S24: Calculated XRD patterns of H_2PTCDI, Me_2PTCDI and Ph_2PTCDI using CuKα radiation at 1.540598 angström.

Electron-hole Transport:

Table S3: Transfer integral t of the HOMO-HOMO interaction and the electron-hole transfer rate k is listed for both directions t_1 and t_2. In addition, the reorganisation energy λ is included:

Crystal	t_1 / meV	t_2 / meV	λ / kJ mol^{-1}	k_1 / s^{-1}	k_2 / s^{-1}
H_2PTCDI	0.8	91.9	24.9	$1.73\ 10^{9}$	$2.26\ 10^{13}$
Me_2PTCDI	3.5	34.2	25.0	$3.26\ 10^{10}$	$3.13\ 10^{12}$
Ph_2PTCDI	1.4	46.8	26.7	$4.01\ 10^{9}$	$4.80\ 10^{12}$

Additional Supporting Literature:

1 D. J. Wales and J. P. K. Doye, *Journal of Physical Chemistry A*, 1997, **101**, 5111–5116.

2 D. Werner, D. H. Apaydin, D. Wielend, K. Geistlinger, W. D. Saputri, U. J. Griesser, E. Drazevic, T. S. Hofer and E. Portenkirchner, *Journal of Physical Chemistry C*, 2021, **125**, 3745–3757.
